# A novel DCSTAMP antagonist impedes preosteoclast fusion via modulation of RAP1B–RAC1-mediated cytoskeletal remodeling

**DOI:** 10.1038/s12276-025-01591-1

**Published:** 2025-12-22

**Authors:** Zheng Zhang, Zhengbo Tao, Weijin Zhang, Zhanrong Zhang, Xuanrui Zhang, Xunpei Xu, Biao Yang, Yichen Meng, Xia Tao, Xuhui Zhou

**Affiliations:** 1 Department of Orthopedics, Changzheng Hospital, Second Military Medical University (Naval Medical University), Shanghai, China; 2 Department of Endocrine and Metabolic Diseases, Shanghai Institute of Endocrine and Metabolic Diseases, Ruijin Hospital, Shanghai Jiao Tong University School of Medicine, Shanghai, China; 3 Shanghai National Clinical Research Center for Metabolic Diseases, Key Laboratory for Endocrine and Metabolic Diseases of the National Health Commission of the People’s Republic of China, Shanghai Key Laboratory for Endocrine Tumor, State Key Laboratory of Medical Genomics, Ruijin Hospital, Shanghai Jiao Tong University School of Medicine, Shanghai, China; 4 Department of Pharmacy, Changzheng Hospital, Second Military Medical University (Naval Medical University), Shanghai, China; 5 Translational Research Center of Orthopedics, Shanghai General Hospital, Shanghai Jiao Tong University School of Medicine, Shanghai, China; 6 Department of Stress Medicine, Faculty of Psychology, Second Military Medical University (Naval Medical University), Shanghai, China

**Keywords:** Actin, Osteoporosis

## Abstract

DCSTAMP serves as a critical fusogenic protein orchestrating cell–cell fusion during osteoclastogenesis. The disruption of DCSTAMP functionality preserves preosteoclasts, thereby augmenting bone mass through both anabolic and anti-catabolic mechanisms. Despite its therapeutic potential, specific DCSTAMP inhibitors remain undiscovered. Here we used structure-based virtual screening utilizing AlphaFold predictions to identify a novel small molecule, E8431, which selectively targets the endoplasmic domain of DCSTAMP. In vitro investigations confirm E8431’s capacity to impede preosteoclast fusion, concurrently inhibiting bone resorption while stimulating PDGFBB secretion, thus promoting osteogenic and angiogenic processes. We further elucidated a previously uncharacterized DCSTAMP signaling cascade involving DCSTAMP–RAP1B interaction, which activates RAP1–RAC1 signaling-dependent cytoskeletal reorganization. Notably, E8431 demonstrates potent inhibitory effects on this DCSTAMP–RAP1B molecular interface. Moreover, E8431 administration effectively attenuates ovariectomy-induced bone loss in murine models without apparent toxicity, underscoring its potential as a therapeutic agent for osteoporosis.

## E8431 targets DCSTAMP to enhance bone mass and health

This study explores how bone health is maintained and how it can be disrupted, leading to conditions such as osteoporosis. Researchers aimed to find a better treatment by targeting a protein called DCSTAMP, which plays a role in bone cell formation. They used advanced computer models to predict the structure of DCSTAMP and identify potential drugs that could inhibit its function. They tested these drugs in lab experiments and found that E8431 effectively reduced the formation of bone-resorbing cells without harming other cells. E8431 also promote bone formation and blood vessel growth, making it a promising candidate for osteoporosis treatment. E8431 could also offer a new way to treat osteoporosis by addressing multiple aspects of bone health. Future research may focus on refining E8431 for clinical use and exploring its long-term effects. This summary was initially drafted using artificial intelligence, then revised and fact-checked by the author.

## Introduction

The human bone undergoes continuous remodeling governed by an intricate equilibrium between osteogenic, osteoclastogenic and angiogenic processes^1^. Pathological conditions, including aging and estrogen deficiency, can disrupt this homeostasis through excessive bone resorption and/or insufficient osteogenesis and microvascular formation, culminating in osteoporosis and elevated fracture risk^2,3^. Although substantial strides have been made in osteoporosis therapeutics, current treatment modalities remain suboptimal^4^. Antiresorptive agents, predominantly bisphosphonates and denosumab (monoclonal antibody of receptor activator of nuclear factor-κB ligand (RANKL)), represent the most frequently prescribed therapeutic interventions for osteoporosis^5^; however, these medications may induce severe adverse events, notably, osteonecrosis of the jaw, albeit with rare occurrence^6^. Furthermore, prolonged bisphosphonate administration may precipitate atypical fractures and femoral head avascular necrosis, whereas denosumab discontinuation triggers rapid, severe bone loss, potentially resulting in multiple vertebral compression fractures^7–9^. In addition, anabolic agents are exemplified by a teriparatide peak during the initial 6–9 months of therapy and then markedly diminish thereafter, limiting their long-term therapeutic utility^10,11^. These limitations of current osteoporosis treatments may stem from their singular focus on specific aspects of bone remodeling rather than addressing the comprehensive regulatory network.

Bone remodeling is initiated by osteoclast-mediated bone resorption followed by osteoblast-mediated bone formation, with the transition phase between these processes being critically regulated by preosteoclasts^12,13^. These tartrate-resistant acid phosphatase (TRAP)-positive, mononuclear cells, characterized by minimal resorptive capacity, undergo DCSTAMP-mediated fusion to generate mature, multinucleated osteoclasts^14,15^. Notably, preosteoclasts secrete platelet-derived growth factor subunit B (PDGFBB), which induces CD31^hi^EMCN^hi^ (type H) vessel formation and recruits osteoprogenitors, ultimately promoting osteogenesis^16^. Consequently, targeting DCSTAMP to prevent preosteoclast fusion represents an auspicious therapeutic paradigm for intervention in pathological bone loss conditions. Previous investigations by our research group have established the efficacy of bone-targeted nanocarrier systems for DCSTAMP small interfering RNA (siRNA) delivery, demonstrating substantial antiosteopenic activity in murine models^17^. Nevertheless, the potential toxicity and immunogenicity associated with RNA–nanoparticle complexes present important barriers to clinical implementation^18–20^. Furthermore, the ligand interactions and endoplasmic signaling cascade of DCSTAMP remain incompletely characterized. This knowledge gap, coupled with the absence of experimentally determined three-dimensional structures, has hindered the development of selective DCSTAMP inhibitors capable of suppressing bone resorption while maintaining preosteoclast populations.

Recent advances in artificial intelligence have revolutionized structural biology^21,22^. DeepMind’s AlphaFold has demonstrated an unprecedented accuracy in predicting protein structures from primary sequences, surpassing traditional methodologies in comprehensive assessments^23^. The recent release of AlphaFold-predicted structures encompassing the entire human proteome has dramatically expanded structural coverage and catalyzed novel approaches in structure-based drug design^24,25^. Virtual screening, using high-throughput molecular docking between protein targets and extensive small molecule libraries, enables the rapid evaluation of millions of potential therapeutic candidates^26,27^. Several pioneering studies have validated the utility of AlphaFold-predicted structures in successful drug development endeavors^28–30^. Therefore, computational screening strategies leveraging AlphaFold-derived structural models offer a compelling approach for the identification of efficacious DCSTAMP-targeted therapeutic agents.

Through the generation of mice lacking the DCSTAMP endoplasmic tail, we demonstrated the essential role of this domain in maintaining DCSTAMP functionality. Subsequently, we used the AlphaFold-predicted DCSTAMP structural models to conduct a virtual screening, culminating in the identification of a specific antagonist targeting endoplasmic region of DCSTAMP. Comprehensive in vitro and in vivo analyses were performed to evaluate this compound’s impact on osteoclastogenesis, osteogenesis and angiogenesis. Finally, we used high-throughput transcriptome sequencing combined with coimmunoprecipitation (Co-IP) and mass spectrometric analyses to delineate the downstream signaling mechanisms of DCSTAMP and its antagonist.

## Materials and methods

### Human bone mineral density association analysis

The UK Biobank estimated bone mineral density (eBMD) genome-wide association study (GWAS) data and human osteoclast expression quantitative trait loci (eQTL) data were obtained from the Genetic Factors for Osteoporosis Consortium (http://www.gefos.org/?q=content/data-release-2018 and http://www.gefos.org/?q=content/human-osteoclast-eqtl-2018-2020) (refs. ^31–33^). Then, the genetic association statistic data of the *DCSTAMP* gene region (approximately ±10 kb of the gene region, hg19, chr11: 105342024-105378917) were analyzed by colocalization with the Coloc2 package^34^.

### Mice

All animal procedures adhered to protocols approved by the Ethics Committee on Animal Experiments of Naval Medical University. The experimental subjects were housed in a specific pathogen-free environment at the Naval Medical University Animal Experimental Center (SYXK 2017-0004) under standardized environmental parameters (24 °C ± 3 °C, 55% ± 5% relative humidity, 12-h light–dark cycle). The rodents were provided a standardized diet comprising corn (40%), bran (25%), bean cake (30%) and supplementary constituents (5%, including mineral salts, bone meal and essential vitamins).

The Dcstamp tail^*−/−*^ mice were generated by CRISPR–Cas9-based genome editing. The expression vectors pCMV-T7-SpRY-P2A-EGFP (RTW4830) (Addgene plasmid no. 139989) and pT7-[BsaI_cassette]-SpCas9_sgRNA_scaffold (MSP3485) (Addgene plasmid no. 140082) were purchased from OBiO. Complementary oligonucleotides (5′-ATAGTGTTGTCTTCTATGCTGATG-3′ and 3′-ACAACAGAAGATACGACTACCAAA-5′) were synthesized by GENEWIZ China and subsequently cloned into pT7-[BsaI_cassette]-SpCas9_sgRNA_scaffold via BsaI restriction digestion and ligation. SpRY Cas9 and single guide RNA (sgRNA) transcripts were generated via in vitro transcription utilizing the Hifair T7 High Yield RNA Synthesis Kit (Yeasen). The cytoplasmic microinjection of the synthesized mRNAs was performed following established protocols^35,36^. The genotypic characterization was accomplished through PCR amplification and Sanger sequencing. The primer sequences and anticipated amplicon sequences for wild-type (WT) and mutant alleles are detailed in Supplementary Tables 1 and 2.

### Mouse models

The ovariectomy (OVX) was performed according to previously established protocols^37^. In brief, following anesthesia administration, the flank region was depilated and aseptically prepared. A lateral incision was made, followed by the meticulous dissection of the musculature to expose and excise the ovaries. Postsurgical recovery proceeded for 4 or 6 weeks, after which the animals were killed for subsequent analyses.

### Human sample

The blood samples were collected from the Department of Orthopedics, Changzheng Hospital, Second Military Medical University (Naval Medical University). All the samples were obtained under the informed or broad consent of volunteers. The study was approved by the ethics committee of the hospital (no. 82372434). The human bone marrow mesenchymal stem cells (hBMSCs) and human umbilical vein endothelial cells (HUVECs) were purchased from OriCell Therapeutics.

### MicroCT

Excised femora were subjected to fixation in 4% paraformaldehyde for 24 h. The morphometric analyses of distal femoral architecture were conducted via high-resolution micro-computed tomography (MicroCT; SkyScan 1076, Bruker) utilizing standardized acquisition parameters (8 μm per pixel resolution, 80-kV voltage, 124-μA current). The comprehensive quantification of trabecular indices, encompassing bone volume fraction (bone volume/total volume (BV/TV), %), trabecular number (1/mm), trabecular separation (mm), trabecular thickness (mm) and trabecular bone mineral density (g/cc), in conjunction with cortical metrics including cortical thickness (mm), was performed using CTAn and CTVol analytical platforms.

### Bone morphology analysis

Murine femora were collected, fixed in 4% paraformaldehyde and subjected to decalcification in 10% EDTA for 3 weeks. Following paraffin embedding, 4-μm sections were prepared for histological analyses, including hematoxylin and eosin (H&E) staining, TRAP staining, osteocalcin (OCN) immunohistochemistry and CD31/endomucin (EMCN) double immunohistochemistry, utilizing previously established protocols^37,38^.

A dynamic histomorphometric analysis utilizing double fluorochrome labeling was conducted to assess bone turnover kinetics, including mineral apposition rate (MAR) and bone formation rate per bone surface (BFR/BS), following established methodologies^37,39^. The image acquisition was performed via inverted microscopy, with subsequent quantification using OsteoMeasure analytical software (OsteoMetrics).

### Flow cytometry

CD31^hi^EMCN^hi^ cell populations in femoral and tibial specimens were quantified via flow cytometric analysis according to established protocols^17,40^. In brief, following bone dissection and bilateral sectioning, specimens were centrifuged at 10,000*g* for 15 s within modified collection tubes to collect whole bone marrow. Post-ACK erythrocyte lysis, cellular suspensions were labeled with a panel of fluorophore-conjugated antibodies: phycoerythrin/Cy7 anti-mouse CD31 (BioLegend), EMCN monoclonal antibody (eBioscience), fluorescein isothiocyanate (FITC) anti-mouse TER-119/erythroid cells (BioLegend) and FITC anti-mouse CD45 (BioLegend). The flow cytometric analysis was performed using a CyAn ADP Analyzer (Beckman Coulter), with bone marrow endothelial cells identified as CD31^+^CD45^−^Ter119^−^ populations.

### Prediction and assessment of DCSTAMP protein structure

Human and mouse DCSTAMP protein sequences and their subcellular localization data were retrieved from the UniProt database (https://www.uniprot.org/). Three-dimensional structural predictions were generated using AlphaFold (https://alphafoldserver.com/) according to standardized protocols^23^. The predicted DCSTAMP structures underwent comprehensive quality assessment utilizing the SWISS-MODEL structure assessment tools (https://swissmodel.expasy.org/assess) and SAVES v6.1 platform (https://saves.mbi.ucla.edu/) to evaluate model confidence and structural validity^41–46^.

### Virtual screening and molecular docking

The DrugBank database (Version 5.1.12), encompassing 13,162 clinical and preclinical small molecular compounds, along with the Enamine screening library comprising premium, functional, advanced and HTS collections (totaling 2,655,449 molecules) were subjected to virtual screening against the human DCSTAMP protein.

The initial molecular docking was conducted utilizing Autodock-Vina in semi-flexible mode, with compounds exhibiting binding affinities below −8.0 kcal/mol selected for subsequent analysis. The secondary screening was performed using Autodock-tools in flexible docking mode, with hits defined by binding energies below −10.0 kcal/mol. Selected compounds were procured from TOPSCIENCE. In addition, the molecular docking of human and mouse DCSTAMP proteins with E8431 were visualized by Pymol v3.1 and LigPlot+ v2.2.

### SPR assay

The surface plasmon resonance (SPR) analyses were conducted at 25 °C utilizing a BIAcore T200 system equipped with CM5 sensor chips according to the manufacturer’s specifications. Human or mouse DCSTAMP protein was covalently immobilized on the CM5 chip surface via amino acid residue conjugation in immobilization buffer. Serial dilutions of E8431 in analyte buffer were introduced to the flow cells to evaluate DCSTAMP-E8431 binding kinetics, comprising 150-s association and 300-s dissociation phases. The kinetic parameters were determined using BIAcore T200 Evaluation software (GE Healthcare).

### RNA-seq and bioinformatic analysis

Primary mouse bone marrow monocytes (mBMMs) derived from WT mice were subjected to M-CSF/RANKL-induced differentiation with or without 2 μM E8431 supplementation, whereas mBMMs from Dcstamp Tail^−/−^ mice underwent M-CSF/RANKL stimulation, both for 5 days. The total RNA was extracted via the TRIzol methodology and subjected to bulk RNA sequencing analysis. Transcriptomic analysis was performed by Shanghai OBiO utilizing the Illumina Novaseq 6000 platform. The raw fastq files underwent initial processing via proprietary perl scripts, followed by paired-end clean read alignment to the reference genome using Hisat2 v2.0.5. StringTie (v1.3.3b) was used for the reference-based assembly of mapped reads, whereas FeatureCounts v1.5.0-p3 quantified gene-mapped read counts for FPKM determination. Differential expression analysis between groups was conducted using the DESeq2 R package, with differentially expressed genes (DEGs) defined by adjusted *P* value <0.05 and log_2_ fold change >0.5. Pathway and process enrichment analyses were performed using the gene ontology (GO), Kyoto Encyclopedia of Genes and Genomes (KEGG) and gene set enrichment analysis (GSEA) frameworks.

### Statistical analysis

The data are presented as mean ± s.d. The normality of distribution was assessed using the Shapiro–Wilk analysis, followed by the *F* test for variance comparison. For normally distributed data with comparable variances, statistical comparisons between two groups were conducted using the two-tailed unpaired Student’s *t*-test, whereas multiple group comparisons used analysis of variance (ANOVA) or ANOVA with Sidak’s multiple comparisons test. Other data underwent nonparametric analyses utilizing the Kruskal–Wallis test for two-group comparisons and Dunn’s multiple comparisons test for multiple groups. The statistical analyses were performed using GraphPad Prism version 8.0 (GraphPad Software) and R software. Statistical significance was denoted as *P < 0.05, **P < 0.01 and ***P < 0.001.

## Results

### Knockout of DCSTAMP endoplasmic tail attenuates OVX-induced bone loss

**Fig. 1:**
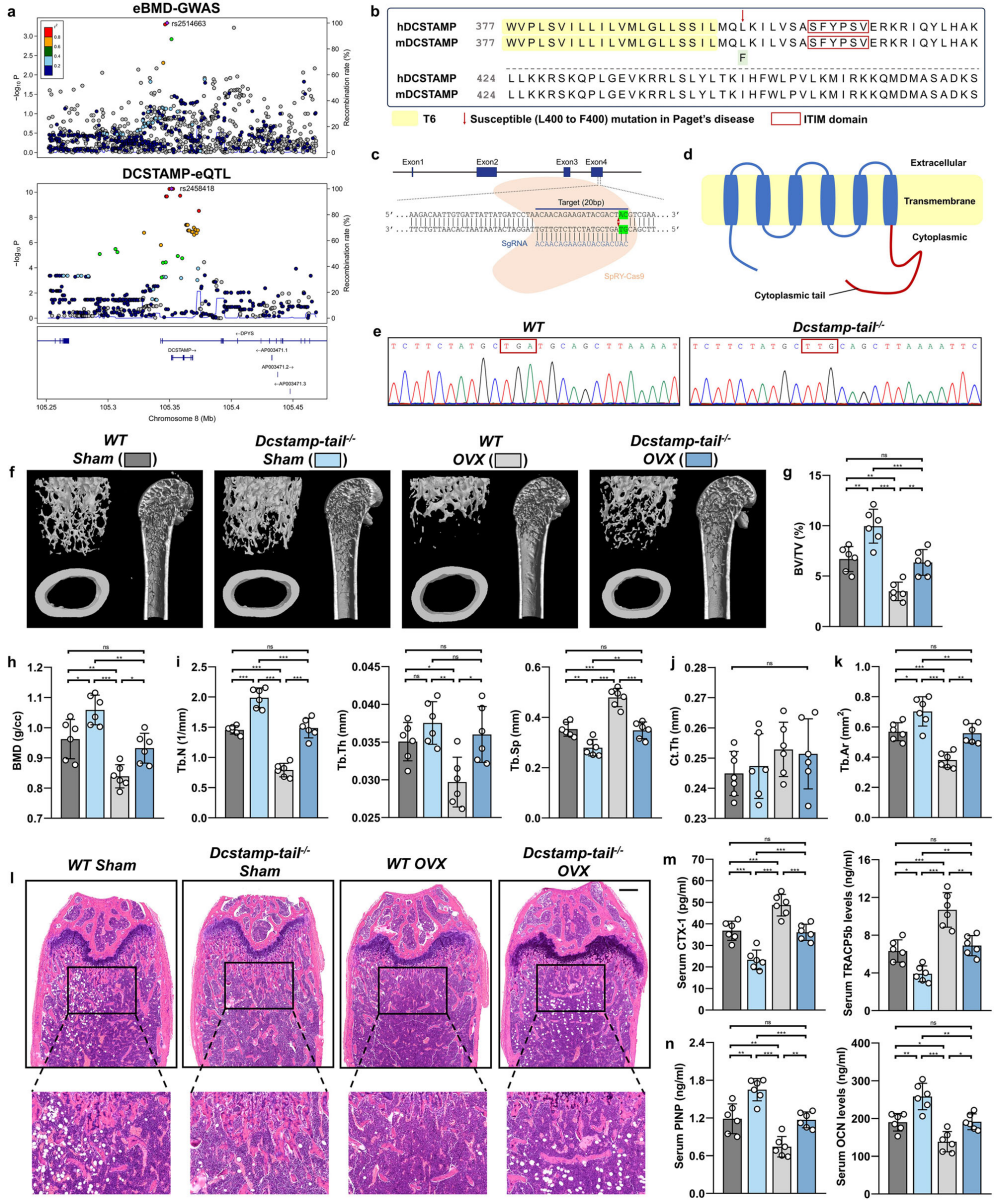
The ablation of the endoplasmic tail of the DCSTAMP protein elicits an augmentation in bone mass in mice. **a** The regional association diagram pertaining to the *DCSTAMP* locus is delineated. The *x* axis denotes the genomic coordinates at chr11: 105342024-105378917 (hg19). Each data point signifies a distinct genetic variant situated within this specific genomic interval. Top: a data point panel exhibiting the correlation of genetic variants with eBMD, with the most statistically significant single-nucleotide polymorphism, rs2514663 (*P* = 4.60 × 10^−4^), highlighted in a regal purple hue. Bottom: a data point panel illustrates the relationship of genetic variants with DCSTAMP expression. The most notable *DCSTAMP* eQTL within osteoclast-like cellular cultures, rs2458418 (*P* = 5.36 × 10^−11^), is also accentuated in a striking purple shade. **b** The sixth transmembrane (T6) segments of the DCSTAMP protein are denoted by a distinct yellow coloration at the base; the single amino acid substitution (L to F) identified in Paget’s disease is indicated by a crimson arrow; the ITIM is signified by a scarlet bracket. **c** The SpRY–Cas9 endonuclease (highlighted in yellow) is precisely directed toward the *DCSTAMP* DNA via an sgRNA encompassing a 20-nucleotide guiding sequence (depicted in blue), leading to a frameshift mutation (displayed in a verdant lower hue). **d** The diagram elucidates that the frameshift mutation results in the inactivation of the cytoplasmic tail of the DCSTAMP protein. **e** The Sanger sequencing of WT and Dcstamp-tail^−/−^ mice. **f** The representative MicroCT analysis of the distal femur is presented for WT mice subjected to sham surgery (WT Sham), DCSTAMP endoplasmic tail knockout mice undergoing Sham surgery (Dcstamp-tail^−/−^ Sham), WT mice undergoing OVX surgery (WT OVX) and DCSTAMP endoplasmic tail knockout mice undergoing OVX surgery (Dcstamp-tail^−/−^ OVX). **g**–**j** Computations derived from MicroCT scans encompassing BV/TV (**g**), bone mineral density (BMD) (**h**), trabecular number (Tb.N), trabecular thickness (Tb.Th), trabecular separation (Tb.Sp) (**i**) and cortical thickness (Ct.Th) (**j**). **k** The enumeration of the Tb.Ar derived from H&E staining. **l** An illustrative representation of H&E staining results from distal femoral sections. Scale bar, 200 μm. **m** The circulating levels of osteoclastic markers, inclusive of CTX-1 and TRACP5b. **n** The circulating levels of osteoblastic markers, encompassing PINP and OCN. *N* = 6 per experimental group. The data are presented as mean ± s.d. with individual data points depicted as dots. Statistical significance levels are denoted as follows: *P < 0.05, **P < 0.01, ***P < 0.001; ns, not significant.

An analysis of genetic summary data encompassing the *DCSTAMP* locus (±10 kb, hg19, chr11: 105342024-105378917) revealed notable associations with eBMD derived from quantitative heel ultrasounds and *DCSTAMP* expression in human osteoclasts. Among 1,202 single-nucleotide polymorphisms within this region, 89 demonstrated remarkable associations with eBMD variations (*P* < 0.05), with rs2514663 emerging as the most significant variant (*P* = 4.60 × 10^−4^), highlighted in purple (Fig. 1a). The integration of GWAS data with eQTL analysis through colocalization established an association between elevated DCSTAMP expression and reduced eBMD (PP.H4 of 0.62) (Fig. 1a).

Previous studies have established the critical role of DCSTAMP’s endoplasmic domain, particularly its tail region (amino acids 398–470), in protein function and osteoclast activity, with a L400F mutation associated with Paget’s disease^47^ (Fig. 1b). To examine the importance of DCSTAMP’s endoplasmic domain integrity, we developed Dcstamp-tail knockout mice (Dcstamp-tail^−/−^) with a deletion of amino acids 398–470 using SpRY–Cas9-mediated frameshift mutagenesis^48,49^ (Fig. 1c–e). A western blot confirmed the selective ablation of DCSTAMP’s C-terminal region while preserving the third extracellular domain, substantiating the successful establishment of this genetically modified murine model (Supplementary Fig. 1).

Male Dcstamp-tail^−/−^ mice showed minimal, statistically insignificant increases in bone mass (Supplementary Fig. 2). Female Dcstamp-tail^−/−^ mice demonstrated significant trabecular bone enhancement compared with sham-operated WT controls, evidenced by increased BV/TV, trabecular bone mineral density, trabecular number, trabecular thickness and decreased trabecular separation (Fig. 1f–j). Moreover, ovariectomized Dcstamp-tail^−/−^ mice exhibited substantial bone mass preservation, achieving levels comparable to sham-operated WT mice (Fig. 1f–j). A histological analysis revealed increased trabecular area (Tb.Ar) in Dcstamp-tail^−/−^ mice under both sham and OVX conditions (Fig. 1k, l). A serum marker analysis showed reduced osteoclastic markers (C-terminal telopeptide of type I collagen (CTX-1) and TRAP isoform 5b (TRACP5b)) and elevated osteoblastic markers (procollagen I N-terminal propeptide (PINP) and OCN) by the knockout of the Dcstamp-tail (Fig. 1m, n). These findings collectively demonstrate that the disruption of DCSTAMP’s endoplasmic domain impairs its function, resulting in enhanced bone mass.

### Dcstamp-tail^−/−^ mice exhibited suppressed osteoclastogenesis and promoted osteogenesis and angiogenesis

**Fig. 2:**
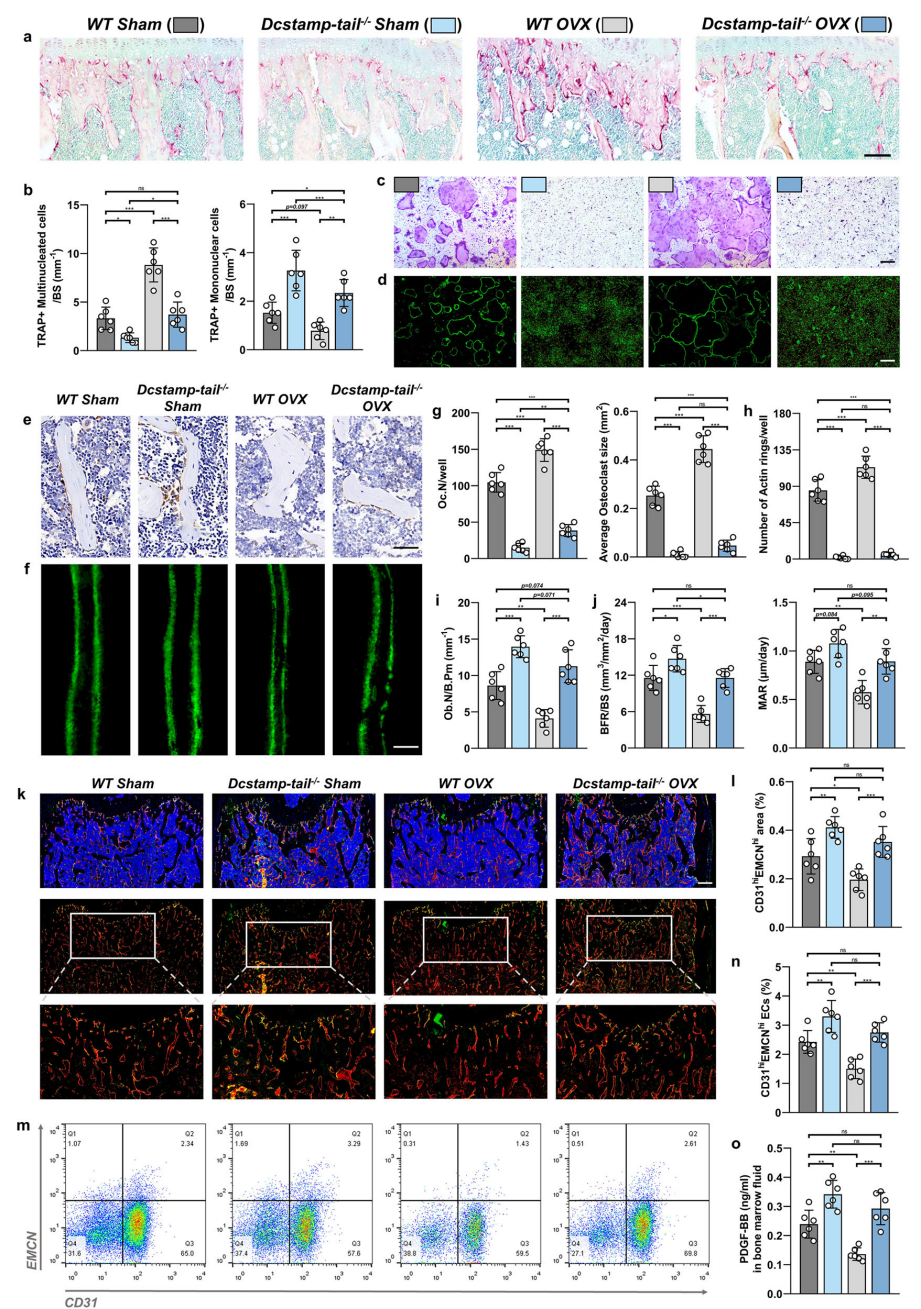
Dcstamp tail^−/−^ mice exhibit diminished bone resorption along with enhanced bone and vasculature formation. **a** A representative TRAP-stained histological sections of distal femora from WT mice subjected to sham operation (WT Sham), DCSTAMP endoplasmic tail-deficient mice with sham surgery (Dcstamp-tail^−/−^ Sham), ovariectomized WT mice (WT OVX) and ovariectomized DCSTAMP endoplasmic tail-deficient mice (Dcstamp-tail^−/−^ OVX). Scale bar, 100 μm. **b** A quantitative analysis of TRAP-positive multinucleated cells (mature osteoclasts) and TRAP-positive mononuclear cells (preosteoclasts) derived from histological sections. **c** The TRAP-positive mature osteoclasts differentiated from primary bone marrow-derived macrophages. Scale bar, 200 μm. **d** A visualization of cytoskeletal architecture utilizing FITC-conjugated phalloidin for F-actin detection. Scale bar, 200 μm. **e** The immunohistochemical analysis of OCN expression in distal femoral sections. Scale bar, 20 μm. **f** The calcein double staining performed on sections of the distal femur. Scale bar, 20 μm. **g** The quantification of osteoclast quantities and average sizes per well garnered from TRAP staining of osteoclasts differentiated in vitro. **h** The quantification of F-actin ring formations derived from FITC-phalloidin staining. **i** The calculation of the osteoblast count per bone perimeter (Ob.N/B.pm) from sections immunohistochemically stained for OCN. **j** A comprehensive quantification analysis of calcein staining results, encompassing BFR/BS and MAR. **k** The depictions of immunostaining showcasing EMCN in red, CD31 in green and CD31^hi^Emcn^hi^ (colored yellow) on trabecular bone. Scale bar, 200 μm. **l** The quantification of the area occupied by CD31^hi^Emcn^hi^ (yellow) cells on trabecular bone based on immunostaining images. **m**, **n** The flow cytometry plots displaying the proportion of CD31^hi^Emcn^hi^ endothelial cells within CD31^+^CD45^−^Ter119^−^ bone marrow cells. **o** The quantification of PDGFBB protein levels in bone marrow assessed via ELISA. *N* = 6 per experimental group. The data are presented as mean ± s.d. with individual data points depicted as dots. Statistical significance levels are denoted as follows: *P < 0.05, **P < 0.01, ***P < 0.001; ns, not significant.

We assessed the impact of DCSTAMP endoplasmic tail deletion on the coupling between osteoclasts, osteoblasts and endothelial cells. TRAP staining revealed reduced numbers of multinucleated TRAP+ cells (mature osteoclasts) in Dcstamp-tail^−/−^ mice compared with WT controls under sham conditions (Fig. 2a, b). Moreover, the OVX-induced increase in multinucleated TRAP+ cells were substantially attenuated in Dcstamp-tail^−/−^ mice (Fig. 2a, b). Conversely, mononuclear TRAP+ cells (preosteoclasts) increased significantly in both sham and OVX conditions (Fig. 2a, b). In vitro osteoclastogenesis assays using primary mBMMs demonstrated that although OVX enhanced mature osteoclast formation in WT cells, Dcstamp-tail^−/−^ mBMMs showed a nearly complete inhibition of mature osteoclast formation in both sham and OVX conditions, as evidenced by TRAP and F-actin staining (Fig. 2c, d, g, h).

A bone morphology analysis revealed that, although OVX reduced OCN^+^ cell (osteoblast) numbers in WT mice compared with sham controls, Dcstamp-tail^−/−^ mice maintained higher OCN^+^ cell counts in both sham and OVX conditions (Fig. 2e, i). The double calcine staining demonstrated that OVX-induced reductions in BFR/BS and MAR were significantly reversed by Dcstamp-tail knockout (Fig. 2f, j). The CD31 and EMCN double immunofluorescence showed expanded type H endothelium in Dcstamp-tail^−/−^ mice, with flow cytometry confirming increased CD31^hi^EMCN^hi^ endothelial cells (Fig. 2k–n). Notably, in vitro osteogenic differentiation of primary mouse bone marrow mesenchymal stem cells (mBMSCs) showed no differences between groups, suggesting that the enhanced in vivo osteogenesis resulted from the altered bone marrow microenvironment (Supplementary Fig. 3). Consistent with this, bone marrow PDGFBB levels were significantly elevated in Dcstamp-tail^−/−^ mice under both sham and OVX conditions (Fig. 2o). The primary mBMMs isolated from male WT and Dcstamp-tail^−/−^ mice demonstrated that Dcstamp-tail deletion also prevented the formation of large multinucleated TRAP^+^ cells (Supplementary Fig. 4a–e). In addition, mBMSCs from male Dcstamp-tail^−^^/−^ mice exhibited no differences in osteogenic or adipogenic differentiation capacity compared with WT controls (Supplementary Fig. 4f–m).

Collectively, Dcstamp-tail deletion inhibits osteoclastogenesis and bone resorption while indirectly enhancing bone formation and type H vessel development through the modulation of the bone microenvironment.

### Virtual screening using AlphaFold3 predicted structure reveals a novel DCSTAMP antagonist

**Fig. 3:**
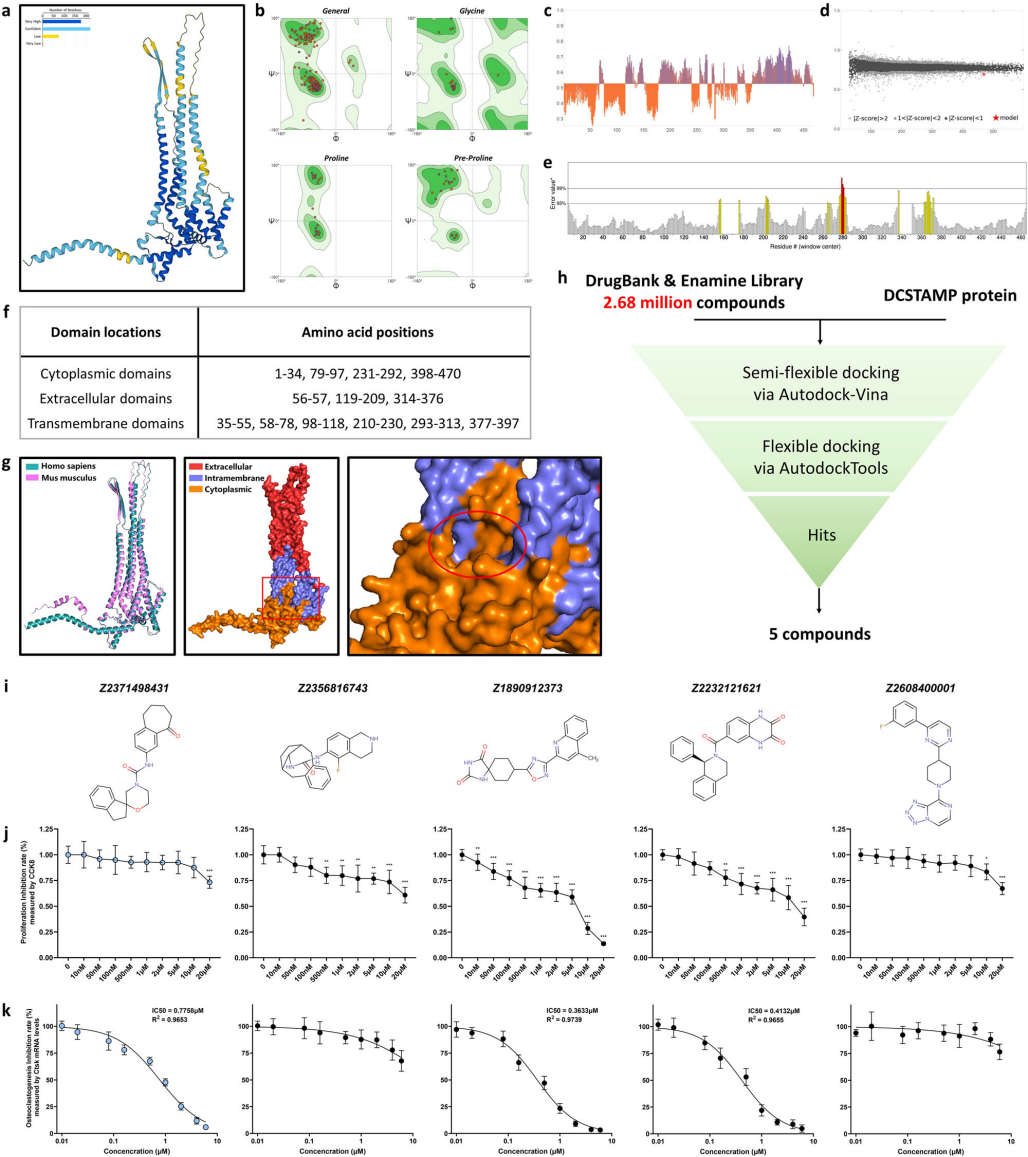
Virtual screening and experimental validation of DCSTAMP antagonists. **a** A representation of the predicted human DCSTAMP protein structure generated by AlphaFold3. **b** A Ramachandran plot illustrating the conformational quality of the human DCSTAMP protein structure using the SWISS-MODEL structure assessment. **c** The QMEAN local scores highlighting the quality of DCSTAMP protein residues in the B-factor column. **d** The comparative analysis with a nonredundant set of PDB structures. **e** An ERRAT analysis demonstrating the percentage of the protein where the calculated error value falls below the 95% rejection limit. **f** An illustration depicting the subcellular distribution of the DCSTAMP sequence. **g** The alignment of predicted human and mouse DCSTAMP proteins along with the subcellular distribution of the human DCSTAMP protein. The red frame indicates a potential active site. **h** The overview of the virtual screening strategy used in this investigation. **i** Chemical structures of the five molecules selected through the screening. **j** An assessment of the proliferation rate of primary mBMMs using a CCK8 assay under various concentrations of the five screened molecules; *N* = 5 per experimental group. **k** The evaluation of the inhibition rate of osteoclastogenesis indicated by Ctsk mRNA levels in primary mBMMs treated with M-CSF/RANKL under varying concentrations of the five screened molecules; *N* = 5 per experimental group. The data are presented as mean ± s.d. Statistical significance levels are denoted as follows: *P < 0.05, **P < 0.01, ***P < 0.001; ns, not significant.

The structures of human and mouse DCSTAMP proteins were predicted using AlphaFold3, with over 80% of residues achieving very high or confident confidence scores (Fig. 3a and Supplementary Fig. 5a). The structure validation through the SWISS-MODEL assessment yielded favorable results: the Ramachandran plot analysis confirmed appropriate torsional angles (*φ* and *ψ*) for most amino acids, and QMEAN analysis produced a global score of 0.53 (human) and 0.61 (mouse), with local scores indicating rational structural predictions compared with existing Protein Data Bank (PDB) structures (Fig. 3b–d and Supplementary Fig. 5b–d). The ERRAT analysis demonstrated that most of the protein structure fell below the 95% rejection limit, with overall quality factors of 94.651 and 96.226 for human and mouse DCSTAMP proteins, respectively (Fig. 3e and Supplementary Fig. 5e). The cytoplasmic, transmembrane and extracellular domains were mapped onto both human and mouse DCSTAMP structures (Fig. 3f). The sequence alignment between human and mouse DCSTAMP revealed high conservation (Fig. 3g). The domain positions and potential active site (highlighted in red) were annotated on the predicted structure (Fig. 3g and Supplementary Fig. 5a).

To identify potential DCSTAMP antagonists, we conducted a comprehensive virtual screening through molecular docking using the Drugbank library (17,425 molecules) and Enamine library (2,665,449 compounds, including functional, premium, advanced and HTS collections). An initial screening using Autodock-Vina in semiflexible mode identified 13,271 compounds with binding affinities below −8.0 kcal/mol. A secondary screening using Autodock-tools in flexible docking mode further selected compounds with binding energies below −10.0 kcal/mol (Fig. 3h). The resulting five compounds were evaluated for their effects on the osteoclastic differentiation of mBMMs (Fig. 3i). Two compounds (Z2356816743 and Z2608400001) failed to inhibit cathepsin K (Ctsk) expression during osteoclastogenesis at concentrations up to 10 μM (Fig. 3i–k). Although Z1890912373 and Z2232121621 effectively suppressed Ctsk expression, their half maximal inhibitory concentrations (IC_50_) showed toxicity toward BMM proliferation (Fig. 3i–k). Only Z2371498431 demonstrated significant osteoclastogenesis inhibition (IC_50_ of 0.7758 μM) without cytotoxic effects on mBMMs at concentrations up to 10 μM (Fig. 3i–k). Consequently, Z2371498431 from the Enamine library was designated as E8431 and selected for further investigation as a novel DCSTAMP antagonist.

### E8431 inhibits osteoclastogenesis in vitro

**Fig. 4:**
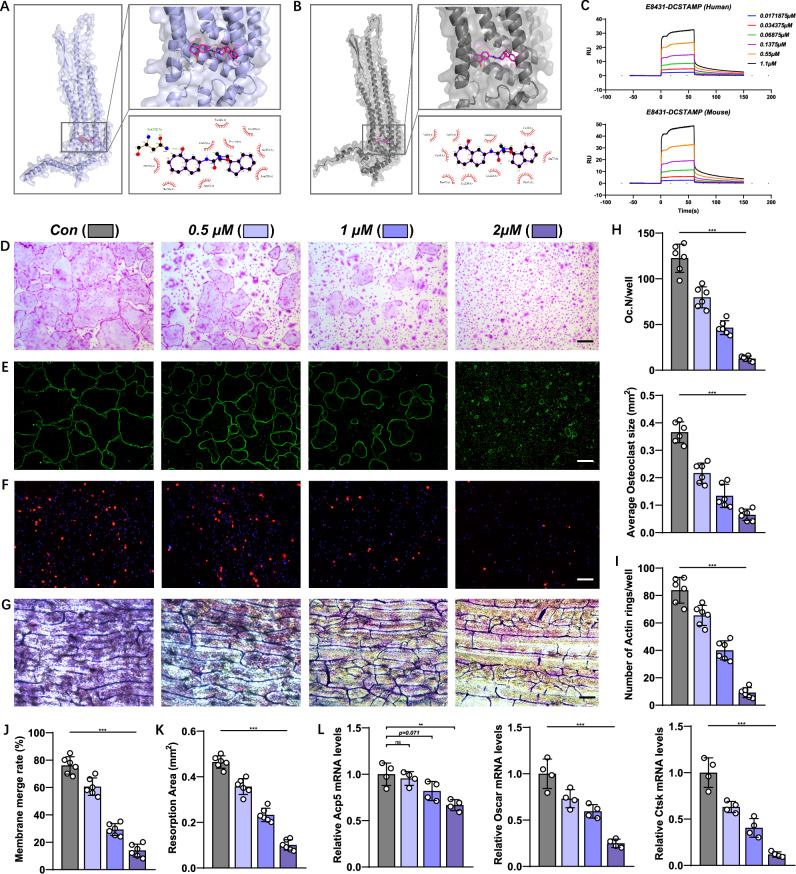
The DCSTAMP antagonist hampers preosteoclast fusion and bone resorption in vitro. **a** A molecular docking visualization illustrating the binding of E8431 to human DCSTAMP. **b** Molecular docking representation showcasing the binding of E8431 to mouse DCSTAMP. **c** Sensorgrams from SPR experiments depicting the interaction between varying concentrations of E8431 and human/mouse DCSTAMP proteins. **d** The TRAP staining displaying mature osteoclasts derived from primary mBMMs. Scale bar, 200 μm. **e** A visualization of the F-actin cytoskeleton via FITC-phalloidin staining. **f** The cell–cell fusion assay conducted with preosteoclasts. Scale bar, 200 μm. **g** A pit formation assay evaluating osteoclast activity. Scale bar, 100 μm. **h** The quantification of osteoclast quantities and average sizes per well derived from the TRAP staining of in vitro differentiated osteoclasts. Scale bar, 200 μm. **i** The quantification of F-actin ring formations based on FITC-phalloidin staining. **j** An assessment of the membrane merge rate from the cell–cell fusion assay. **k** The quantification of the resorption area from the pit formation assay. **l** An evaluation of the transcriptional expression levels of osteoclastic genes such as Acp5, Oscar and Ctsk. *N* = 4 for qPCR assays per group; *N* = 6 for other panels. The data are presented as mean ± s.d. Statistical significance levels are denoted as follows: *P < 0.05, **P < 0.01, ***P < 0.001; ns, not significant.

The flexible molecular docking analysis revealed E8431 binding to human and mouse DCSTAMP proteins with binding energies of −10.4 kcal/mol and −10.1 kcal/mol, respectively (Fig. 4a, b). Moreover, the deletion of the DCSTAMP endoplasmic tail domain abolishes the molecular interaction between E8431 and both human and murine DCSTAMP protein variants. The SPR assay further validated the direct binding interaction between E8431 and both human and mouse DCSTAMP proteins, confirming its role as a DCSTAMP antagonist (Fig. 4c). Conversely, upon the ablation of the intracellular domain of DCSTAMP, both human and murine DCSTAMP proteins exhibited a complete inability to associate with E8431, thus abrogating ligand–receptor interaction (Supplementary Fig. 6).

E8431’s effects on osteoclast differentiation, fusion and function were evaluated through in vitro experiments using mBMMs. The TRAP staining revealed the dose-dependent inhibition of mature osteoclast number and size, with 2 μM E8431 showing maximal efficacy (Fig. 4d, h). The E8431 treatment also reduced the number of functional osteoclasts, as evidenced by decreased actin-ring formation (Fig. 4e, i). The osteoclast fusion assay demonstrated significant reduction in preosteoclast membrane fusion rates, whereas the pit formation assay showed decreased osteoclast resorption area following E8431 treatment (Fig. 4f, g, j, k). The gene expression analysis showed that acid phosphatase 5, tartrate resistant (Acp5), the common marker for osteoclast/preosteoclast, remained largely unchanged at 0.5 μM and 1 μM E8431, with modest reduction to 75% of control levels at 2 μM (Fig. 4l). However, mature osteoclast-specific markers, including osteoclast associated Ig-like receptor (OSCAR) and Ctsk, were significantly and dose-dependently downregulated across all tested concentrations (0.5, 1 and 2 μM) (Fig. 4l).

To validate E8431’s effects in human cells, peripheral blood mononuclear cells (PBMCs) isolated from healthy donors were subjected to osteoclast differentiation assays with and without E8431 treatment. The CCK8 assay confirmed no remarkable cytotoxicity at concentrations up to 20 μM (Supplementary Fig. 7a). TRAP staining, F-actin staining and assessment of osteoclast markers (OSCAR and CTSK) demonstrated notable reduction in the formation of large, mature and functional osteoclasts from human PBMCs (Supplementary Fig. 8).

These findings demonstrate that E8431 inhibits osteoclast fusion and resorption through binding with DCSTAMP protein. The optimal E8431 treatment concentration was determined to be approximately 2 μM, at which osteoclast numbers were reduced to 10–20% of control levels without exhibiting substantial toxicity.

### E8431 promotes angiogenesis in the coculture system of mEPCs with mBMMs in vitro

We first treated mouse primary mouse endothelial progenitor cells (mEPCs) with various concentrations of E8431, which shows E8431 under the concentration of 10 μM having no significant cytotoxicity (Supplementary Fig. 7b). In addition, the treatment of 0.5 or 2 μM E8431 showed no effects on mEPCs’ migration and tube formation (Supplementary Fig. 9).

**Fig. 5:**
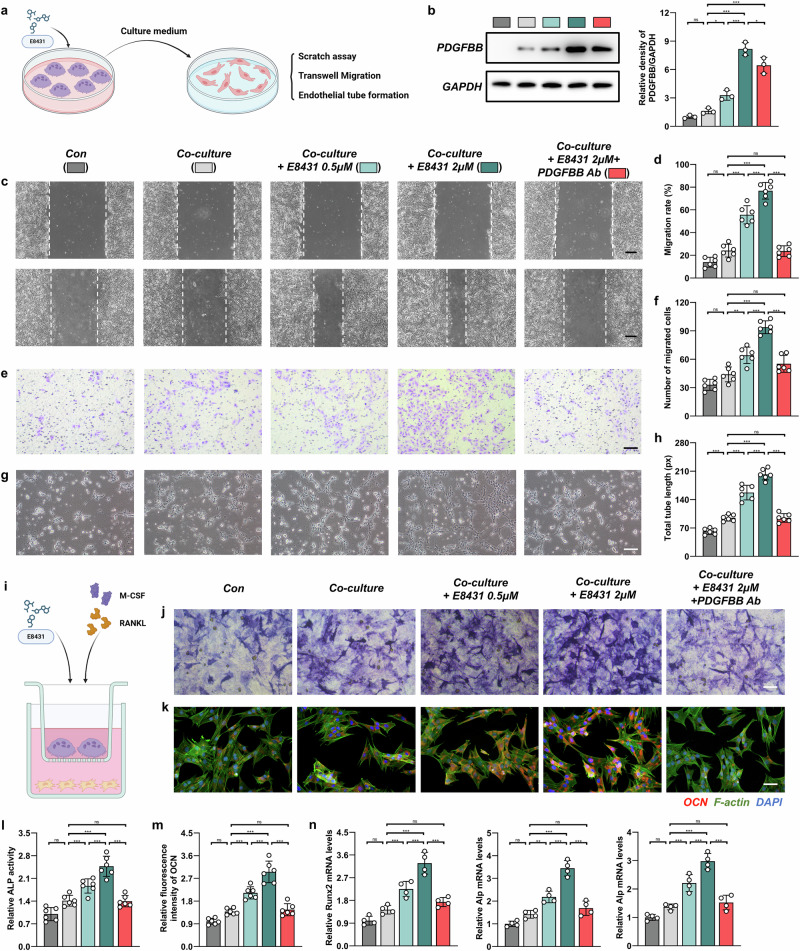
The DCSTAMP antagonist potentiates osteogenic and angiogenic processes through inhibitory modulation of PDGFBB secretion from pre-osteoclastic fusion events in vitro. **a** A demonstration of the coculture of mEPCs and mBMMs under M-CSF/RANKL stimulation with or without E8431. **b** A western blot analysis quantifying the levels of PDGFBB protein in mEPCs. **c** Representative images of mEPC scratch wound assay at 0 h (upper panel) and 24 h (lower panel). Scale bar, 200 μm. **d** Quantification of migration rate from scratch assay. **e** Representative images of mEPC migration through Transwell chambers. Scale bar, 200 μm. **f** Quantification of migrated cell number from Transwell assay. **g** Representative images of mEPC tube formation on Matrigel. Scale bar, 200 μm. **h** Quantification of total tube length from mEPC tube formation assay. **i** A depiction of the coculture of mBMSCs and mBMMs in an osteogenic culture medium under M-CSF/RANKL stimulation with or without E8431. **j** The ALP staining of mBMSCs. Scale bar, 25 μm. **k** An immunofluorescence visualization of OCN in mBMSCs. Scale bar, 25 μm. **l** The quantification of ALP activity. **m** The quantification of OCN fluorescence intensity from OCN immunofluorescence. **n** The relative mRNA expression levels of osteogenic markers including Runx2, Alp and Bglap. The experimental conditions were systematically organized as follows: Con, endothelial progenitor cells (EPCs)/bone marrow mesenchymal stem cells (BMSCs) maintained without intervention; coculture, EPCs/BMSCs cocultivated with mBMMs absent additional treatments; coculture + E8431 0.5 μM, EPCs/BMSCs cocultivated with mBMMs supplemented with E8431 at 0.5 μM concentration; coculture + E8431 2 μM: EPCs/BMSCs cocultivated with mBMMs supplemented with E8431 at 2 μM concentration; coculture + E8431 2 μM + PDGFBB Ab, EPCs/BMSCs cocultivated with mBMMs supplemented with both E8431 (2 μM) and PDGFBB neutralizing antibody (100 μg/ml). *N* = 4 in western blot and qPCR assays per group; *N* = 6 in other panels. The data are presented as mean ± s.d. with individual data points depicted as dots. Statistical significance levels are denoted as follows: *P < 0.05, **P < 0.01, ***P < 0.001; ns, not significant. Parts **a** and **i** created with BioRender.com.

To elucidate whether the E8431-mediated inhibition of DCSTAMP-dependent osteoclast fusion preserves preosteoclasts and subsequently enhances angiogenesis via PDGFBB secretion, we established a coculture system comprising mesenchymal stem cells/mEPCs and osteoclasts under E8431 administration (Fig. 5a). The western blot analysis revealed elevated PDGFBB concentrations in primary mBMMs across experimental groups, as illustrated in Fig. 5b, with E8431 treatment substantially augmenting PDGFBB levels. We subsequently investigated the platelet-derived growth factor receptor beta (PDGFRβ)-mediated phosphatidylinositol 3-kinase (PI3K)–AKT serine/threonine kinase (Akt) signaling cascade within the initial hour following the coculture establishment. The E8431-supplemented coculture system exhibited markedly augmented phosphorylation profiles of PDGFRβ, PI3K, Akt and focal adhesion kinase (FAK) (Supplementary Fig. 10). To further elucidate the pivotal role of PDGFBB, we used a PDGFBB neutralizing antibody at a concentration of 100 μg/ml—sufficient to substantially attenuate PDGFRβ activation without inducing appreciable cytotoxicity—which was subsequently utilized as a PDGFBB inhibitor throughout ensuing experimental investigations (Supplementary Fig. 11).

Subsequently, we collected conditioned media from osteoclast cultures, both E8431-treated and untreated, and combined it with standard mEPC culture medium at a 1:2 ratio for subsequent mEPC analyses. Scratch and Transwell migration assays demonstrated that supplementation with conditioned media from both untreated and E8431-treated (0.5 μM/2 μM) osteoclast cultures significantly enhanced mEPC migratory capacity compared with controls (Fig. 5c–f). Notably, 2 μM E8431 treatment yielded the most pronounced enhancement, markedly surpassing the untreated condition (Fig. 5c–f). The tube formation assays further corroborated that osteoclast-conditioned medium promoted mEPC tubulogenesis, with E8431 treatment, particularly at 2 μM, substantially amplifying this effect (Fig. 5g, h). Furthermore, the introduction of the PDGFBB neutralizing antibody to the coculture system completely abolished the proangiogenic effects mediated by E8431 in vitro (Fig. 5a–h).

To enhance the translational potential of E8431, we evaluated its effects on HUVECs. E8431 exhibited no cytotoxicity at concentrations up to 20 μM, and the direct application of 0.5 μM E8431 had no significant impact on HUVECs’ migration or tube formation (Supplementary Figs. 7c and 12a–d). Upon coculture with E8431-treated osteoclasts, HUVECs demonstrated notably enhanced migratory capacity and tubulogenesis (Supplementary Fig. 12e–h). These findings collectively demonstrate that E8431 augments mEPC migration and tube formation through the preservation of preosteoclast-derived PDGFBB.

### E8431 promotes osteogenesis in the coculture system of mesenchymal stem cells with mBMMs in vitro

We then investigated the influence of E8431 on osteogenic differentiation. Primary mBMSCs collected from murine specimens underwent osteogenic induction in the presence or absence of E8431 supplementation. Notably, E8431 exhibited no inhibitory effects on mBMSC proliferation at concentrations up to 10 μM, whereas osteogenic potential, as quantified by Alizarin Red S and alkaline phosphatase (ALP) staining, remained unperturbed at both 0.5 μM and 2 μM concentrations (Supplementary Figs. 7d and 13). Moreover, the immunoblot analysis demonstrated that the PI3K–Akt–FAK signaling cascade, activated via the PDGFBB receptor engagement, was exclusively phosphorylated under conditions in which both the coculture system and E8431 treatment were simultaneously present (Supplementary Fig. 14).

Subsequently, a coculture system was established utilizing Transwell chambers (0.4 μM), with mBMMs and mBMSCs seeded in the upper and lower compartments, respectively (Fig. 5i). E8431 was administered at concentrations of 0.5 μM and 2 μM. The ALP staining revealed significantly enhanced ALP activity in cocultured groups compared with controls, with E8431 treatment, particularly at 2 μM, markedly augmenting this effect (Fig. 5j, l). The immunofluorescence analysis of OCN expression corroborated that E8431-treated coculture systems substantially elevated OCN levels in mBMSCs (Fig. 5k, m). Moreover, expression of osteogenic marker genes, including RUNX family transcription factor 2 (Runx2), Alp and bone gamma-carboxyglutamate protein (Bglap, gene encoding OCN), was substantially upregulated in E8431-treated co-culture conditions (Fig. 5n). Concurrently, the incorporation of PDGFBB neutralizing antibody into the co-culture system entirely nullified the pro-osteogenic effects conferred by E8431 in vitro (Fig. 5i–n).

In addition, we explored E8431’s effects on human BMSCs (hBMSCs). E8431 demonstrated a favorable safety profile in hBMSCs up to 20 M, with no discernible impact on osteogenic differentiation at 0.5 μM or 2 μM, as assessed by Alizarin Red S and ALP staining (Supplementary Figs. 7e and 15a–e). In a Transwell coculture system comprising hBMSCs and PBMCs, the E8431 treatment significantly enhanced hBMSC osteogenic differentiation, as evidenced by increased ALP activity (Supplementary Fig. 15f, g).

These findings demonstrate that E8431 augments hBMSC osteogenesis through the preservation of preosteoclasts and subsequent activation of PDGFBB-mediated PI3K–FAK signaling cascade.

### E8431 inhibits OVX-induced bone deterioration

We then sought to evaluate E8431’s efficacy in mitigating bone loss in vivo. Following the intraperitoneal administration of E8431 at 20 mg/kg and 100 mg/kg, the serum concentrations were monitored at 1, 4, 8, 12, 18 and 24 h post injection. The 20 mg/kg dose yielded a peak concentration of 1.227 mg/l (3.141 μM), declining below the therapeutic threshold within approximately 4 h, rendering it insufficient for treatment (Supplementary Fig. 16). By contrast, the 100 mg/kg dose achieved a peak concentration of 3.582 mg/l (9.173 μM), maintaining levels above the therapeutic threshold for approximately 16 h, thus establishing it as the appropriate dosage for subsequent in vivo studies (Supplementary Fig. 16).

Subsequently, an ovariectomized mouse model was established, followed by the daily intraperitoneal administration of 100 mg/kg E8431. Following 6 weeks of treatment, a serum analysis for cardiac (creatine kinase and lactate dehydrogenase), hepatic (alanine aminotransferase and aspartate transaminase) and renal (creatinine) injury markers revealed no significant alterations (Supplementary Fig. 17a–c). Furthermore, a histological examination of major organs (heart, lung, liver, spleen and kidney) by H&E staining demonstrated no appreciable pathological changes in response to E8431 treatment (Supplementary Fig. 17d).

**Fig. 6:**
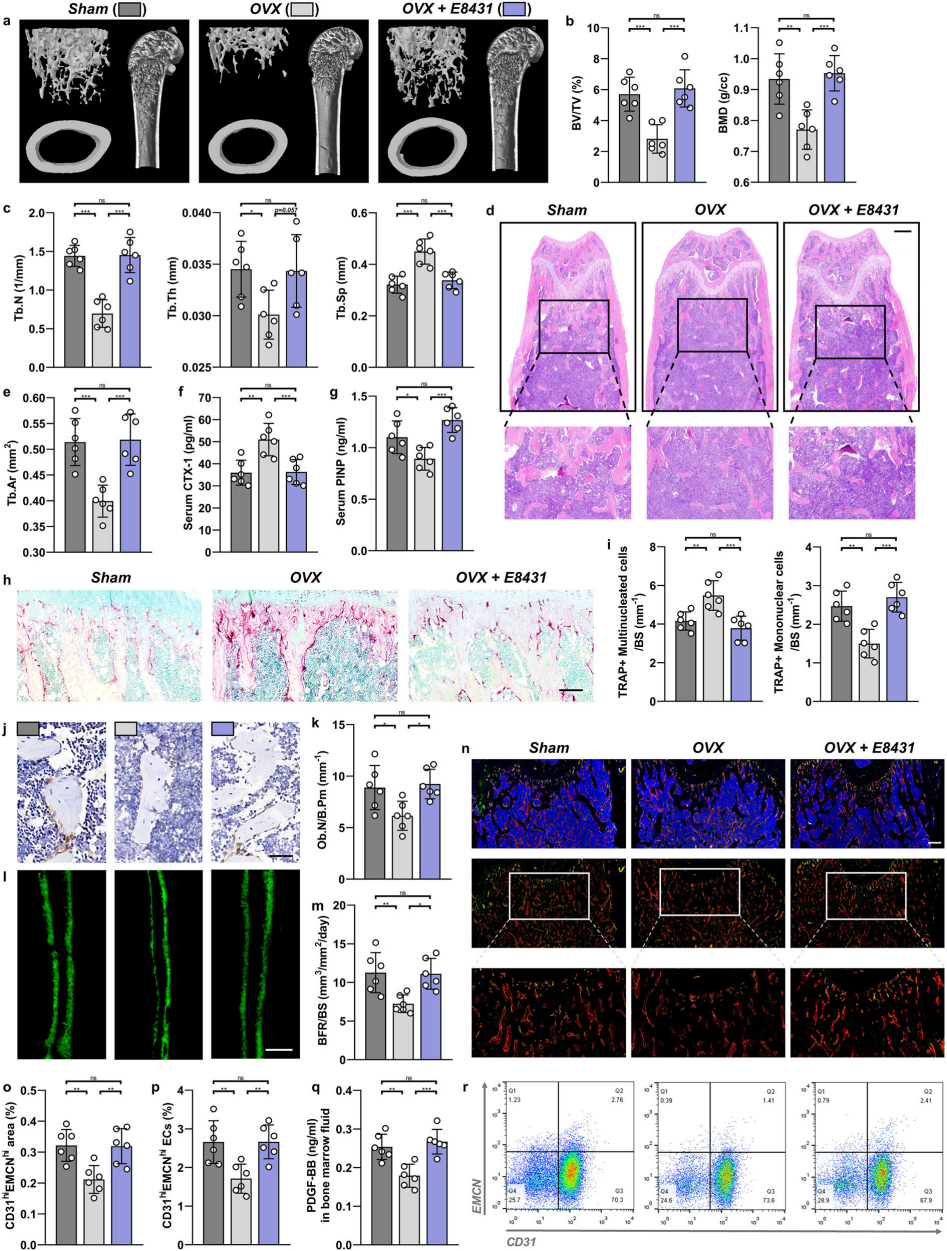
The DCSTAMP antagonist mitigates bone loss resulting from estrogen deficiency in mice. **a** A representative MicroCT evaluation of the distal femur in murine subjects subjected to sham surgery (WT), OVX and OVX with E8431 intervention (OVX + E8431). **b**, **c** Measurements derived from MicroCT scans including BV/TV, BMD (**b**) trabecular number (Tb.N), trabecular thickness (Tb.Th) and trabecular separation (Tb.Sp) (**c**). **d** An illustrative H&E staining of distal femoral sections. Scale bar, 200 μm. **e** The quantification of the Tb.Ar from H&E staining. **f**, **g** The serum levels of osteoclastic marker CTX-1 (**f**) and osteoblastic marker PINP (**g**). **h** The representative sections of the distal femur stained for TRAP. Scale bar, 100 μm. **i** The quantification of TRAP-stained multinuclear cells (mature osteoclasts) and TRAP-positive mononuclear cells (preosteoclasts). **j** The OCN immunohistochemistry sections of the distal femur. Scale bar, 20 μm. **k** The quantification of osteoblast count per bone perimeter (Ob.N/B.pm) from OCN immunohistochemical sections. **l** The calcein staining of distal femoral sections. **m** The quantitative analysis of calcein staining, indicating BFR/BS. Scale bar, 20 μm. **n** The immunostaining images showing EMCN (red), CD31 (green) and CD31^hi^EMCN^hi^ (yellow) cells on the trabecular bone. Scale bar, 200 μm. **o** The quantification of the CD31^hi^EMCN^hi^ (yellow) cell area on the trabecular bone from immunostaining images. **p** Representative flow cytometry plots showing CD31^hi^EMCN^hi^ endothelial cells within the CD31^+^CD45^−^Ter119^−^ bone marrow cell population. **r** Quantification of CD31^hi^EMCN^hi^ endothelial cell percentage from flow cytometry. **q** The measurement of the protein levels of PDGFBB in bone marrow using ELISA. *N* = 6 per experimental group. The data are presented as mean ± s.d. with individual data points depicted as dots. Statistical significance levels are denoted as follows: *P < 0.05, **P < 0.01, ***P < 0.001; ns, not significant.

A MicroCT analysis of the distal femur revealed that the E8431 administration completely reversed OVX-induced bone loss, demonstrating substantial improvements in BV/TV, BMD, trabecular number and trabecular thickness while reducing trabecular separation (Fig. 6a–c). Concordantly, a histological examination showed that the E8431 treatment restored OVX-diminished Tb.Ar to levels comparable to the sham group (Fig. 6d, e). Furthermore, E8431 normalized serum markers of bone turnover, including CTX-1 and PINP, indicating its dual regulatory effects on osteogenesis and osteoclastogenesis (Fig. 6f, g).

The osteoclast activity assessment by TRAP staining revealed that E8431 reduced TRAP-positive multinucleated cells (mature osteoclasts) while increasing TRAP-positive mononuclear cells (preosteoclasts) (Fig. 6h, i). OCN-positive cell populations were notably expanded following E8431 administration under OVX conditions (Fig. 6j, k). The dynamic histomorphometry using double calcein labeling demonstrated that E8431 restored the OVX-suppressed bone formation rate (Fig. 6l, m). Type H endothelium, visualized through CD31 and EMCN coimmunofluorescence and quantified by flow cytometry, showed notable recovery from OVX-induced reduction following E8431 treatment (Fig. 6n–p, r). The enzyme-linked immunosorbent assay (ELISA) analysis confirmed elevated bone marrow PDGFBB levels in E8431-treated OVX conditions (Fig. 6q). Taken together, these findings establish E8431 as an efficacious therapeutic agent for osteopenia.

### Dcstamp-tail knockout and E8431 inhibits osteoclastogenesis via RAP1 signaling

**Fig. 7:**
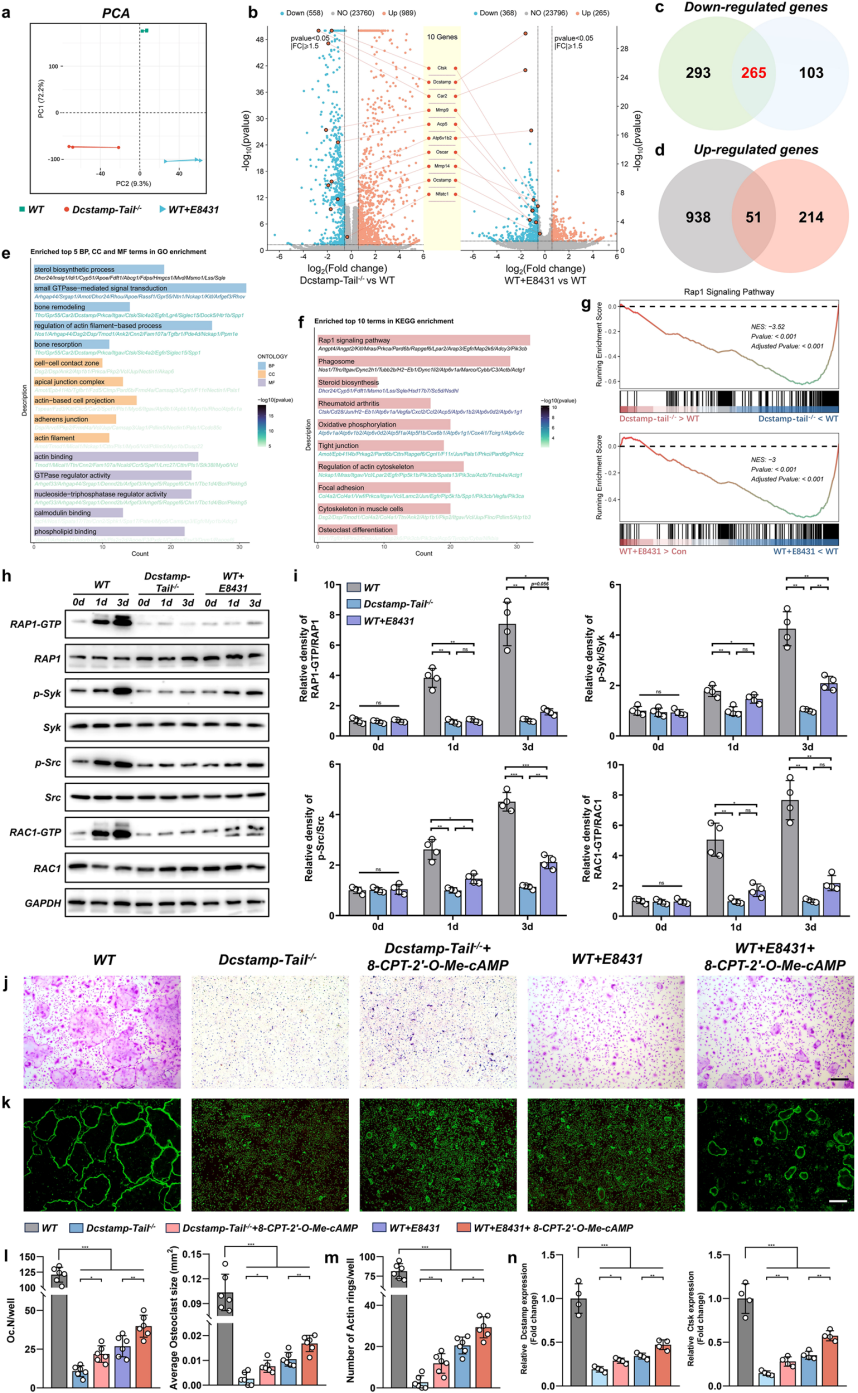
Interference with DCSTAMP function results in the disruption of RAP1–RAC1 signaling pathways during osteoclastogenesis. **a** A principal component analysis conducted on mature osteoclasts derived from primary mBMMs obtained from WT, Dcstamp tail^−/−^ and WT mice treated with E8431. **b** Volcano plots illustrating the DEGs between the Dcstamp tail^−/−^ and WT groups, as well as the WT + E8431 and WT groups. Each group consisted of *N* = 3 samples. Key osteoclast marker genes, such as Ctsk, Dcstamp, Car2, Mmp9, Acp5, Atp6v1b2, Oscar, Mmp14, Ocstamp and Nfatc1, are highlighted. **c**, **d** Venn diagram representations delineating the shared downregulated genes (**c**) and upregulated genes (**d**) in the Dcstamp tail^−/−^ and WT + E8431 groups compared with the WT group. **e** The GO analysis performed on the commonly downregulated genes in the Dcstamp tail^−/−^ and WT + E8431 groups compared with the WT group. **f** A KEGG analysis conducted on the commonly downregulated genes in the Dcstamp tail^−/−^ and WT + E8431 groups compared with the WT group. **g** A GSEA examining the Rap1 signaling pathway in the comparison between the Dcstamp tail^−/−^ and WT groups, as well as the WT + E8431 and WT groups. **h** A western blot analysis measuring the protein levels of RAP1-GTP, RAP1, phosphorylated Syk (p-Syk), Syk, phosphorylated Src (p-Src), Src, RAC1-GTP, RAC1 and GAPDH. **i** The quantification of the western blot results, with *N* = 4 samples analyzed. **j** The TRAP staining mature osteoclasts differentiated from primary mBMMs of different groups including WT, Dcstamp-tail^−/−^, Dcstamp-tail^−/− ^+ 8-CPT-2′-O-Me-cAMP, WT + E8431 and WT + E8431 + 8-CPT-2′-O-Me-cAMP. **k** The visualization of F-actin cytoskeleton via the FITC-phalloidin staining of different groups. **l** The quantification of osteoclast numbers and average size per well from TRAP staining of in vitro differentiated osteoclasts. *N* = 6 per group. **m** The quantification of F-actin rings from FITC-phalloidin staining. *N* = 6 per group. **n** The transcriptional expression levels of osteoclastic genes including Dcstamp and Ctsk. *N* = 4 per group. The data are presented as mean ± s.d. with individual data points depicted as dots. Statistical significance levels are denoted as follows: *P < 0.05, **P < 0.01, ***P < 0.001; ns, not significant.

We further investigated the molecular mechanisms underlying E8431’s inhibition of DCSTAMP function. Primary mBMMs isolated from WT, Dcstamp-tail^−/−^ and E8431-treated WT mice underwent osteoclastic differentiation followed by RNA sequencing analysis. Principal component analysis revealed that the transcriptional profile of E8431-treated WT cells more closely resembled Dcstamp-tail^−/−^ cells than WT controls (Fig. 7a). A differential gene expression analysis comparing Dcstamp-tail^−/−^ versus WT and E8431-treated WT versus WT groups is presented in Fig. 7b. Both Dcstamp-tail^−/−^ and E8431-treated groups exhibited the downregulation of key osteoclast markers, including Ctsk, Dcstamp, carbonic anhydrase 2 (Car2), matrix metallopeptidase 9 (Mmp9), ATPase H^+^ transporting v1 subunit b2 (Atp6v1b2), Oscar, matrix metallopeptidase 14 (Mmp14) and osteoclast stimulatory transmembrane protein (Ocstamp) (Fig. 7b). Notably, Nfatc1 expression remained unchanged, suggesting that the DCSTAMP endoplasmic domain does not critically regulate Nfatc1 expression (Fig. 7b). The overlapping downregulated genes constituted approximately 50% of all downregulated genes in the Dcstamp-tail^−/−^ group and two thirds in the E8431-treated group, indicating substantial mechanistic convergence (Fig. 7c). By contrast, upregulated genes showed less concordance between Dcstamp-tail^−/−^ and E8431-treated groups (Fig. 7d).

We performed enrichment analysis on the shared downregulated genes between Dcstamp-tail^−/−^ and E8431-treated groups. The GO analysis revealed remarkable enrichment in bone remodeling and resorption pathways, alongside GTPase-mediated signal transduction and actin filament-associated processes (Fig. 7e). The KEGG pathway analysis identified Rap1 signaling as the most significantly regulated pathway among the common downregulated genes, with additional substantial alterations in actin cytoskeleton regulation and osteoclast differentiation pathways (Fig. 7f). Given previous evidence establishing RAP1–RAC1 signaling as a critical mediator of cytoskeletal remodeling and osteoclast differentiation, we investigated whether the RAP1–RAC1 cascade represents a key downstream effector of DCSTAMP signaling.

The GSEA demonstrated the significant downregulation of the Rap1 signaling pathway in both Dcstamp-tail^−/−^ and E8431-treated groups (Fig. 7g). During osteoclastogenesis, RAP1 and RAC1 activation states and phosphorylation of spleen-associated tyrosine kinase (Syk) and proto-oncogene tyrosine-protein kinase SRC (Src) were markedly elevated; however, these effects were substantially attenuated by both Dcstamp-tail knockout and E8431 treatment (Fig. 7h, i). In rescue experiments utilizing the Rap1 agonist 8-CPT-2′-O-Me-cAMP, the western blot analysis revealed that RAP1–RAC1 signaling activation was restored, partially rescuing the impaired mature osteoclast formation observed in both Dcstamp-tail knockout and E8431-treated conditions (Fig. 7j–n and Supplementary Fig. 18). These findings establish RAP1–RAC1 signaling and associated cytoskeletal remodeling as critical downstream effectors of DCSTAMP-mediated signaling.

### Dcstamp-tail knockout and E8431 directly disrupts the binding of DCSTAMP and RAP1B

**Fig. 8:**
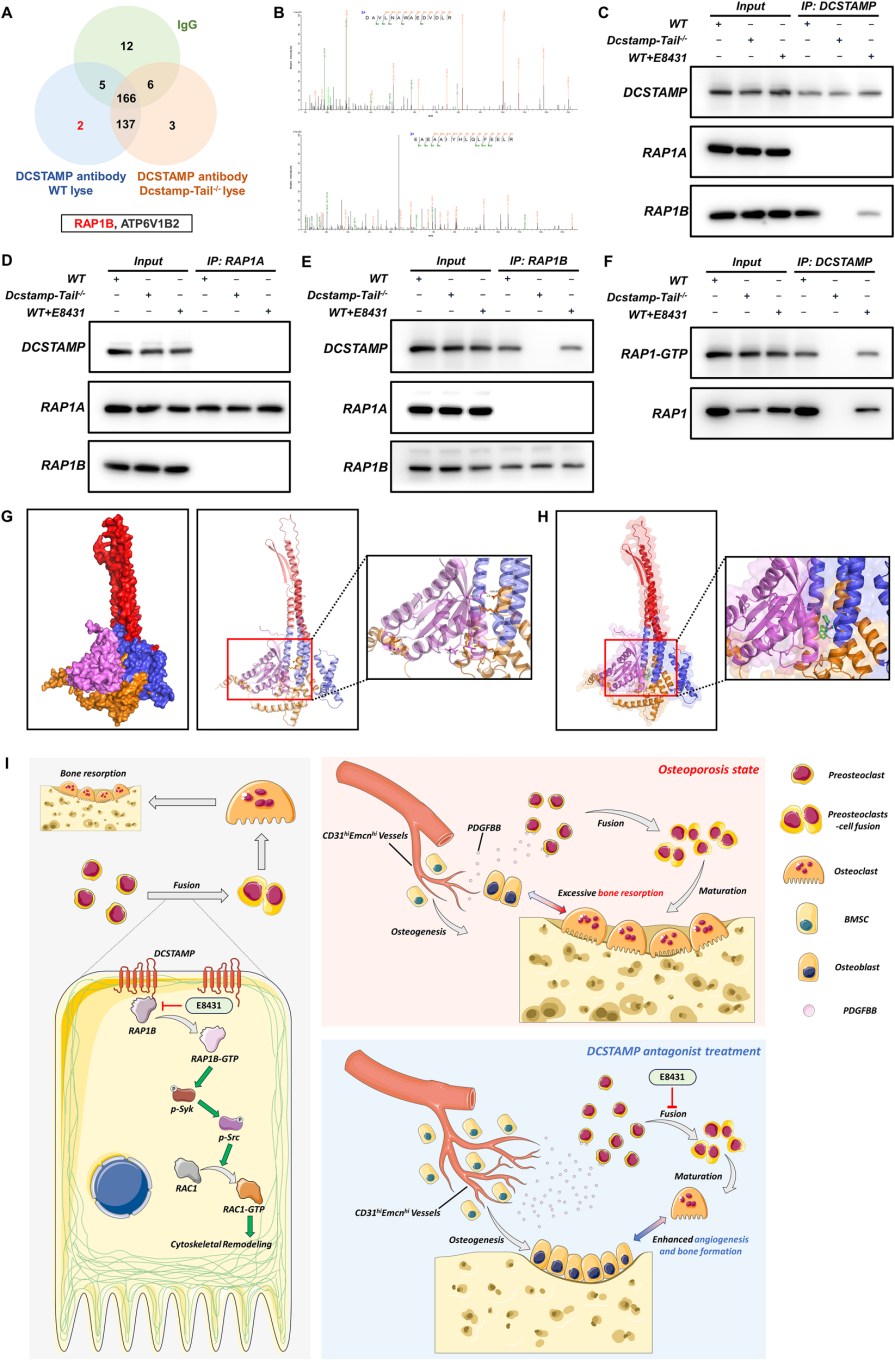
DCSTAMP antagonist hinders the interaction between DCSTAMP and RAP1B, thereby impeding cytoskeletal remodeling and osteoclastogenesis. **a** The proteins engaged in the interaction with the DCSTAMP protein in WT and Dcstamp-tail^−/−^ lysates were discerned through mass spectrometry subsequent to RNA pulldown in mBMMs. **b** The presence of the RAP1B protein was ascertained via mass spectrometry analysis. **c** The Co-IP was conducted using an anti-DCSTAMP antibody with WT, Dcstamp-tail^−/−^ and WT + E8431 lysates. **d** The Co-IP was performed using an anti-RAP1A antibody with WT, Dcstamp-tail^−/−^ and WT + E8431 lysates. **e** The Co-IP was carried out utilizing an anti-RAP1B antibody with WT, Dcstamp-tail^−/−^ and WT + E8431 lysates. **f** The levels of RAP1-GTP and RAP1 within the proteins obtained via Co-IP in WT, Dcstamp-tail^−/−^ and WT + E8431 lysates were evaluated. **g** Molecular docking simulations unveiled the interaction between RAP1B and murine DCSTAMP. **h** The molecular docking simulations demonstrated the impact of E8431 on the binding between RAP1B and murine DCSTAMP. **i** A schematic illustration depicting E8431’s molecular mechanisms in regulating DCSTAMP functionality and skeletal homeostasis. E8431 orchestrates the potent inhibition of osteoclastogenesis through the disruption of DCSTAMP–RAP1B molecular complexes, consequently attenuating RAP1–RAC1-dependent cytoskeletal remodeling essential for osteoclast maturation and cellular fusion. Moreover, E8431 intervention markedly ameliorates OVX-induced skeletal deterioration via the preservation of preosteoclast populations, culminating in suppressed bone resorption along with enhanced osteogenesis and neovascularization. The data are presented as mean ± s.d. with individual data points depicted as dots. Statistical significance levels are denoted as follows: *P < 0.05, **P < 0.01, ***P < 0.001; ns, not significant.

To elucidate the mechanism by which DCSTAMP regulates RAP1–RAC1 signaling, we performed Co-IP assays using DCSTAMP antibody in WT and Dcstamp-tail^−/−^ lysates (Fig. 8a). Notably, RAP1B was detected in the immunoprecipitates from WT but not Dcstamp-tail^−/−^ lysates, suggesting the direct regulation of RAP1–RAC1 signaling (Fig. 8a, b). The protein abundance of total RAP1A and RAP1B remained relatively unaltered throughout osteoclastogenic differentiation (Supplementary Fig. 19).

Further Co-IP analyses revealed that, although RAP1A showed no interaction with either DCSTAMP or RAP1B, RAP1B demonstrated robust binding to DCSTAMP under WT conditions, which was remarkably diminished by both Dcstamp-tail knockout and E8431 treatment (Fig. 8c). Reciprocal Co-IP experiments using RAP1A antibody confirmed the absence of interaction with DCSTAMP and RAP1B, whereas RAP1B immunoprecipitation further validated its direct interaction with DCSTAMP (Fig. 8d, e). Moreover, the analysis of pulled-down proteins using the Active Rap1 Detection Kit demonstrated that DCSTAMP-bound RAP1 exhibited notable activation, which was completely abolished by Dcstamp-tail knockout and substantially reduced by E8431 treatment (Fig. 8f).

Molecular docking analysis, illustrated in Fig. 8g, revealed the interaction between RAP1B and the endoplasmic domain of DCSTAMP. E8431 was predicted to occupy the active site of DCSTAMP at the interface between these two proteins, probably disrupting their interaction (Fig. 8h). Collectively, these findings demonstrate that DCSTAMP activates downstream RAP1–RAC1 signaling through direct binding and activation of RAP1B, a process that can be effectively inhibited by either the deletion of the DCSTAMP endoplasmic domain or E8431 treatment (Fig. 8i).

## Discussion

The targeted deletion of DCSTAMP’s endoplasmic tail in murine models elicited enhanced trabecular bone mass and conferred protection against OVX-induced skeletal deterioration. Through structure-based virtual screening of AlphaFold-predicted DCSTAMP conformations, we identified a novel antagonist, E8431, which demonstrated robust therapeutic efficacy against osteopenia by inhibiting preosteoclast fusion while simultaneously promoting osteogenic and angiogenic processes. Mechanistic analyses revealed that E8431 disrupts the interactions between the endoplasmic domain of DCSTAMP and RAP1B, thereby attenuating RAP1–RAC1 signaling-mediated cytoskeletal reorganization crucial for osteoclast multinucleation.

DCSTAMP, a highly conserved protein predominantly expressed in myeloid dendritic cells, macrophages and osteoclasts, exhibits distinct cell type-specific functions, with notably elevated expression during osteoclastogenesis^50,51^. The genetic ablation of Dcstamp in murine models results in impaired multinuclear osteoclast formation both in vivo and in vitro, manifesting as mild osteopetrosis^52–54^. Studies utilizing human PBMCs revealed that DCSTAMP-high cells demonstrate elevated osteoclastogenic potential compared with DCSTAMP-low populations, establishing DCSTAMP as a crucial mediator of cell–cell fusion during osteoclast differentiation^55^. An analysis of CD14^+^ monocytes isolated from 49 healthy female donors revealed age-dependent increases in osteoclast maturation in vitro, with DCSTAMP expression levels exhibiting positive correlations with osteoclast nucleation capacity, serum CTX levels and donor age, thus illustrating DCSTAMP as an important regulator of age-associated pathological alterations in osteoclast function^56^. Our current investigation using Mendelian randomization revealed a potential inverse correlation between human osteoclast DCSTAMP expression and eBMD, further demonstrating DCSTAMP as a therapeutic candidate for osteoporosis intervention.

The endoplasmic domain of DCSTAMP plays an indispensable role in its fusogenic function. The presence of an immunoreceptor tyrosine-based inhibitory motif (ITIM) within the cytoplasmic tail is critical, as its deletion impairs multinuclear osteoclast formation in vitro^54^. In addition, GWASs have identified a significant correlation between Paget’s disease pathogenesis and a leucine-to-phenylalanine substitution at position 400 within the DCSTAMP endoplasmic tail^57,58^. To elucidate the functional significance of the DCSTAMP endoplasmic domain and identify potential therapeutic binding sites, we generated a murine model harboring a targeted deletion of the DCSTAMP endoplasmic tail. Although 12-week-old male Dcstamp-tail^−/−^ mice exhibited unremarkable skeletal phenotypes, potentially attributable to their intrinsically high bone mass, the female counterparts manifested substantial augmentation in trabecular bone density under both physiological and OVX conditions. Notably, OVX Dcstamp-tail^−/−^ mice maintained bone parameters comparable to sham-operated WT controls. Histological analyses further revealed that DCSTAMP tail deletion not only reduced mature osteoclast numbers but also enhanced bone formation and type H vessel development.

Previous investigations have recognized DCSTAMP’s therapeutic potential. Chiu and colleagues generated monoclonal antibody 1A2 directed against residues 447–460 of DCSTAMP, demonstrating the suppression of osteoclast multinucleation in human PBMC cultures^55,59^. However, the intracellular positioning of the target epitope presents a fundamental impediment to therapeutic efficacy, and no in vivo validation data were reported in this study. Dou et al. identified miR-7b as a negative regulator of osteoclastogenesis through DCSTAMP mRNA degradation and developed a polyetherimide-functionalized graphene oxide delivery system for miR-7b overexpression^60,61^. Although this approach mitigated OVX-induced bone loss, the absence of biodistribution and toxicology data, coupled with nontargeted delivery necessitating high dosing, raises critical health implication. Our previous work established a bone-targeted nanoparticle system comprising alendronate-modified polyethylene glycol shells for controlled Dcstamp siRNA delivery^17^. This platform effectively ameliorated estrogen deficiency-induced bone loss while enhancing osteogenic and angiogenic processes, demonstrating no overt organ toxicity during a 6-week treatment regimen^17^. However, the observed hepatic accumulation, an intrinsic limitation of nonviral delivery systems, presents potential long-term safety concerns^62^. Consequently, despite the challenges posed by limited structural data, the development of selective small molecule DCSTAMP inhibitors represents a particularly attractive therapeutic approach.

AlphaFold, trained on multiple sequence alignments and experimental protein structures from the PDB database, achieves structural prediction accuracy comparable to experimental methods^23,63^. With more than 90% of its predicted human transmembrane proteome structures displaying membrane protein-like characteristics, the platform demonstrates substantial potential for virtual screening applications targeting transmembrane proteins^64,65^. Despite ongoing debates regarding the reliability of AlphaFold-predicted structures for ligand design, several pioneering studies have successfully identified specific inhibitors for various enzymes and GPCRs^28,29,65–68^. In our study, AlphaFold-generated human and mouse DCSTAMP structures, validated through SWISS-MODEL and ERRAT analyses, served as templates for virtual screening of more than 2.6 million compounds using Autodock-Vina and Autodock-tools, yielding five potential DCSTAMP inhibitors. In vitro validation revealed that exclusively E8431 exhibited antiosteoclastogenic activity at concentrations that preserved normal cell proliferation, warranting further in vitro and in vivo investigation. These findings support the viability of AlphaFold-based virtual screening as a promising strategy for drug development.

A key innovation of this study was the identification and validation of E8431 as an effective and safe DCSTAMP antagonist. E8431 demonstrated a favorable safety profile, with concentrations up to 10 μM showing no effect on primary BMM, mBMSC and mEPC proliferation, whereas concentrations up to 20 μM preserved human PBMC, hBMSC and hUVEC viability. At 2 μM, E8431 effectively suppressed osteoclastogenesis in both mBMMs and human PBMCs. Although E8431 did not directly influence osteogenesis or angiogenesis, conditioned medium from E8431-treated (2 μM) M-CSF/RANKL-stimulated mBMMs enhanced the osteogenic differentiation of mBMSCs and promoted mEPC migration and tube formation. Similar effects were observed in human cell systems using PBMCs with hBMSCs/HUVECs. The daily intraperitoneal administration of E8431 (100 mg/kg) in OVX mice completely prevented estrogen deficiency-induced bone loss through inhibiting osteoclastogenesis while promoting bone formation and vascularization, without apparent toxicity. These observations underscore the therapeutic promise of E8431 as a bifunctional agent possessing both anabolic and anti-catabolic properties.

The complete mechanisms governing DCSTAMP-mediated preosteoclast fusion remain largely undefined, particularly regarding its endogenous ligand^69^. Several studies have begun to elucidate the endoplasmic signaling cascade downstream of DCSTAMP. In human PBMCs, DCSTAMP undergoes tyrosine phosphorylation and forms physical complexes with both src homology region 2 domain-containing phosphatase 1 (SHP-1) and CD16, potentially integrating with the immunoreceptor tyrosine-based activation motif immunoglobulin and ITIM signaling network during osteoclastogenesis^55^. Furthermore, DCSTAMP inhibition via miR-7b has been shown to suppress c-fos and Nfatc1 expression within this network, thereby inhibiting osteoclastogenesis^47,60^. However, a critical unresolved question concerns DCSTAMP’s role in cytoskeletal rearrangement during repeated cell–cell fusion events, particularly regarding actin-dependent signaling networks^70–72^. Through comparative RNA sequencing analysis of WT, Dcstamp-tail^−/−^ and E8431-treated samples, we identified a remarkable downregulation of actin filament organization, GTPase regulation and Rap1 signaling following the disruption of DCSTAMP’s endoplasmic function. The western blot analysis confirmed the reduced activation of RAP1, Syk, Src and RAC1 in response to both Dcstamp-tail knockout and E8431 treatment. Notably, 8-CPPT-2′-O-Me-cAMP-mediated RAP1 hyperactivation partially rescued the impaired osteoclast and actin-ring formation in both Dcstamp-tail^−/−^ and E8431-treated conditions. Co-IP studies revealed specific binding between RAP1B (but not RAP1A) and DCSTAMP’s endoplasmic domain, an interaction disrupted by E8431. These findings provide the first evidence that DCSTAMP directly modulates cytoskeletal remodeling during osteoclastogenesis through regulation of RAP1–RAC1 signaling.

Several limitations of this study warrant consideration. First, concerns regarding the accuracy of virtual screening using AlphaFold-predicted structures compared with experimentally determined structures may have resulted in overlooking several potential inhibitors. Second, E8431 has not undergone chemical optimization to enhance its DCSTAMP inhibitory activity. In addition, although E8431’s interaction with DCSTAMP has been confirmed, the possibility of interactions with other molecular targets cannot be definitively excluded. Nevertheless, E8431 demonstrated robust efficacy in preventing bone loss in OVX mice, with serum markers and histological analyses of major organs indicating no significant toxicity. Furthermore, E8431’s effectiveness in human cell models, including PBMCs, hMSCs and HUVECs, suggests promising therapeutic potential for clinical translation.

In summary, we identified a novel antagonist, E8431, that targets the endoplasmic domain of DCSTAMP. E8431 effectively suppresses osteoclastogenesis by disrupting DCSTAMP–RAP1B interactions, thereby inhibiting RAP1–RAC1 signaling-mediated cytoskeletal reorganization required for osteoclast maturation and fusion. Moreover, E8431 enhances osteogenesis and angiogenesis through increased PDGFBB secretion from preserved preosteoclasts. Furthermore, E8431 administration substantially attenuates OVX-induced bone loss, demonstrating its therapeutic potential for osteoporosis treatment.

## Supplementary information

Supplementary Information

## Data Availability

The RNA sequencing and mass spectrum data that support the findings of this study are available from the corresponding author upon reasonable request.
