# Supplementary material for: A novel DCSTAMP antagonist impedes preosteoclast fusion via modulation of RAP1B–RAC1-mediated cytoskeletal remodeling
Source: Exp Mol Med. 2025 Dec 22;57(12):2898–915. doi: 10.1038/s12276-025-01591-1 (PMC12800066; doi:10.1038/s12276-025-01591-1)
Supplement: Supplementary file 1 — Supplementary Information [file 12276_2025_1591_MOESM1_ESM.pdf]

Supplemental Material

A novel DCSTAMP antagonist impedes preosteoclast fusion via modulation of RAP1B-RAC1 mediated cytoskeletal remodeling

Zheng Zhang[1,7], Zhengbo Tao[1,7], Weijin Zhang[1,7], Zhanrong Zhang[1], Xuanrui Zhang[1], Xunpei Xu[1], Biao Yang[1], Yichen Meng[1,2,3,*], Xia Tao[4,*], Xuhui Zhou[1,5,6,*]

1. Department of Orthopedics, Changzheng Hospital, Second Military Medical University (Naval Medical University), Shanghai, China

2. Department of Endocrine and Metabolic Diseases, Shanghai Institute of Endocrine and Metabolic Diseases, Ruijin Hospital, Shanghai Jiao Tong University School of Medicine, Shanghai, China

3. Shanghai National Clinical Research Center for Metabolic Diseases, Key Laboratory for Endocrine and Metabolic Diseases of the National Health Commission of the PR China, Shanghai Key Laboratory for Endocrine Tumor, State Key Laboratory of Medical Genomics, Ruijin Hospital, Shanghai Jiao Tong University School of Medicine, Shanghai, China

4. Department of Pharmacy, Changzheng Hospital, Second Military Medical University (Naval Medical University), Shanghai, China

5. Translational Research Center of Orthopedics, Shanghai General Hospital, Shanghai Jiao Tong University School of Medicine, Shanghai, China

6. Department of Stress Medicine, Faculty of Psychology, Second Military Medical University (Naval Medical University), Shanghai, China.

7. These authors contributed equally

Supplementary Materials

Primary murine mBMMs isolation

Osteoclastic differentiation of primary murine mBMMs was conducted following established protocols[1]. Femora and tibiae were harvested from 6-week-old mice, cleaned, and bilaterally sectioned prior to centrifugation at 10,000 ×g for 15 seconds at ambient temperature to extract bone marrow. The resultant marrow suspension was cultured in standard medium for 24 hours, after which non-adherent cells were collected and subjected to Ammonium-Chloride-Potassium (ACK) erythrocyte lysis. Subsequently, cells were seeded in 24-well or 12-well plates for further assays.

Primary mBMSCs isolation

Primary mBMSCs were isolated from femurs and tibias of 6-week-old mice. The isolation of bone marrow from femur and tibia were reported as above. The resultant marrow suspension was cultured in standard medium for 48h to make all mBMSCs adherent to the culture dish. Then remove all non-adherent cells, and culture the adherent cells for more 8-10 days until they reach the density of about 80%. Then the cells were harvested for further assays.

Primary mEPCs isolation

Primary mEPCs were isolated from femurs and tibias of 6-week-old mice. The isolation of bone marrow from femur and tibia were reported as above. Bone marrow was subjected to centrifugation at 1000rpm for 5 min. Then the cell pellet was resuspended by PBS, slowly dropped into the upper layer of Ficoll(Solarbio,P8900), and subjected to density gradient

centrifugation at 2000rpm for 20 minutes. The cells in middle layer were cultured in EBM-2 medium, supplemented with 2%FBS and EGF for 48h. Subsequently, non-adherent cells were removed and adherent cells were collected for further assays.

Primary human PBMCs isolation

Osteoclastic differentiation of primary human monocytes was conducted as previously described[1]. PMBCs were isolated from the blood of participants via density gradient centrifugation. The blood was diluted with an equal volume of phosphate-buffered saline (PBS). The mixture was slowly dropped into the upper layer of Ficoll(Solarbio,P8900) and subjected to density gradient centrifugation at 800g for 20 minutes. Then the cells layer was diluted with PBS and subjected to centrifugation at 300g for 10 minutes twice. The remnant was resuspended by α-MEM medium and cultured in 10-cm plate.

In vitro osteoclastogenesis assay

Cells were cultured in osteoclastogenic medium supplemented with macrophage colony-stimulating factor (M-CSF, 30 ng/mL) and Receptor activator of nuclear factor kappa-B ligand (RANKL, 80 ng/mL) for 5 days.

In vitro osteogenesis and adipogenesis assay

For osteogenic differentiation, BMSCs were seeded in 12-well plates. When the density reached 60–70%, cells were cultured in osteogenic medium (DMEM with 10% FBS, 1% Penicillin/Streptomycin, 5mM β-glycerophosphate, 50 μM ascorbic acid and 100 nM

dexamethasone) for 14 days.

For adipogenic induction, BMSCs were seeded and cultured in 12-well plates until reaching the density of 90-100%. Then the culture medium was replaced by adipogenic medium (α-MEM supplemented with 10% FBS, 1% Penicillin/Streptomycin, 0.5 mM 3-isobutyl-1-methylxanthine, 5 μg/ml insulin, and 1 μM dexamethasone) for 10 days.

CCK-8 assay

For CCK-8 assay, human BMCs were seeded onto 96-well plates at a density of 1000/well and cultured in normal medium for 24h to adhere. Then cells were cultured in different conditions for 24h and 48h. CCK-8 reagent was added into the medium with a ratio 1:10 and cells were incubated in 37°C for 2h to test the cell viability. 450 nm absorbance was measured by the microplate reader.

ARS staining

ARS staining was carried out to assess the osteogenesis of BMSCs. Briefly, cells were fixed in 4% PFA for 20 min. Then BMSCs were stained by 1% ARS (A5533, Sigma-Aldrich Co. Ltd) solution for 15 min. Then they were incubated in 100 mM cetylpyridinium chloride (C0732, Sigma-Aldrich Co. Ltd) for quantitative analysis of the mineralized matrix. After one-hour incubation, the calcium concentration was assessed by spectrophotometer at 562nm.

ALP staining

For ALP staining, cells were fixed in 4% PFA for 20 min and applied ALP staining solution

(Beyotime,C3206), following the instructions provided by the manufacturer. Cells were incubated at 37°C for 30 minutes in the dark. Pictures were taken by a light microscope and evaluated by Image-Pro Plus software.

OCN immunostaining

Tissues were embedded in paraffin and made into sections of 5-µm thickness. Sections were deparaffinized in xylene, rehydrated in graded ethanol. For antigen retrieval, slides were submerged in the citrate buffer (pH 6.0) at 95°C for 30 minutes. Then tissue sections were incubated in blocking buffer (5%BSA in PBS) for 1h at room temperature, applied with the primary OCN antibody with a ratio according to the manufacturer's instruction and incubated in 4°C overnight. After washed with TBS, slides were applied with secondary antibody conjugated with HRP with a ratio according to the manufacturer's instruction and incubated for 1h at room temperature. Slides were examined under a light microscope.

Oil red O staining

Oil red O staining was performed to evaluate the adipogenesis of BMSCs. In brief, cells were fixed in 4% PFA for 20 min and then stained with oil red O (O0625, Sigma-Aldrich Co. Ltd) dye liquor for 20 min. To quantify the oil droplets, 10 pictures were randomly taken by an optical microscope for every well and density (cells per mm2) were calculated.

TRAP staining

TRAP staining was conducted using TRAP staining kit, as instructed by the manufacturer

(Sigma-Aldrich Co. Ltd). Briefly, cells were fixed in 4% PFA for 20 min and subsequently stained by TRAP staining solution for 30 min at 37°C. Pictures were taken by an optical microscope and analyzed by Image-Pro Plus software.

Scratch experiment

Scratch wound assay was performed as previously reported. Briefly, cells were plated in 6-well plates at a density of 5 × 105/well and cultured until reaching the confluence of 100%. Then a p200 pipet tip was used to create a scratch of the cell monolayer. Cells were then cultured in normal medium for another 24h. Photos of the scratches were taken immediately and 24h after the wound were made by microscopy.

Migration experiment

For the transwell migration assay, mEPCs or hUVECs were seeded into the top chamber of 8 μm pore-size transwell plate at a density of 1.5 × 104/well and cultured under different conditions. After 24h, migrated cells were stained with crystal violet and quantified under a microscope.

Tube formation experiment

Cells were seeded in matrigel-coated 12-well plates. Cells were cultured in different conditions for 4h. Then the images of tube formation were obtained by microscopy and cumulative tube length was analyzed by Image-Pro Plus 6 software.

In vitro osteoclast fusion and function test

Osteoclast fusion assay was carried out as previously reported. Briefly, cells were induced by M-CSF (30 ng/mL) and RANKL (80 ng/mL) for 3 days and then labeled with Hoechst or CM-Dil for 20 minutes at room temperature. Then two groups of cells were cultured together for 12 hours and photos were taken by fluorescence microscopy. Detailed analysis of pit formation area was described previously. To observe resorption pits on substrates, cells were fixed by 4% paraformaldehyde and stained with toluidine blue.

Coculture of mBMMs and mBMSCs

Primary mBMMs and Primary mBMSCs were isolated from bone marrow as we described before. Then primary mBMMs and primary mBMSCs were seeded in the top and bottom chamber of 12-well 0.4 μm transwell plate and cultured in osteogenic medium (DMEM medium containing 10 mM β-glycerophosphate, 50 μM ascorbic acid and 100 nM dexamethasone) with or without M-CSF (30 ng/mL) and RANKL (50 ng/mL) for 6 days. Then cells were harvested for different experiments.

Coculture of mBMMs and mEPCs

The mEPCs was acquired from Changzheng hospital and cultured in DMEM basic medium. The mBMMs were cultured in α-MEM medium (with 30 ng/mL M-SCF and 80 ng/mL RANKL) or normal medium for 5 days. The E8431 was added on day2 of osteoclastogenesis induction. Then we collected the osteoclast medium and mixed with normal culture medium at a ratio of 1/2 as conditioned medium (CM) to culture mEPCs for further investigations.

Coculture of PBMCs and hBMSCs

Primary PBMCs and Primary hBMSCs were isolated from bone marrow as we described before. PBMCs and hBMSCs were seeded in the upper and lower transwell chamber, cultured in osteogenic medium (DMEM medium containing 10 mM β-glycerophosphate, 50 μM ascorbic acid and 100 nM dexamethasone) with or without M-CSF (30 ng/mL) and RANKL (50 ng/mL) for 6 days. The PBMCs in the upper chambers were removed, and the hBMSCs in the lower chambers were harvest for other experiments.

Coculture of PBMCs and hUVECs

PBMCs were culture in osteoclastogenic medium for 5 days. The E8431 was added on day2 of osteoclastogenesis induction. HUVECs were cultured in the mixture of the collected osteoclastogenesis medium and ECM complete medium with a ration 1:2. HUVECs were harvested for further assay.

qRT-PCR

Total RNA was isolated from cellular and tissue specimens utilizing RNA quick purification kit and reverse transcribed to cDNA using HiScript III RT SuperMix. Quantitative PCR analyses were performed using Taq Pro Universal SYBR qPCR Master Mix, with transcript levels normalized to Gapdh or GAPDH expression. Target-specific primers were designed in accordance with MIQE guidelines (Supplementary Table 3). Amplification and detection were conducted on a LineGene 9600 Plus (BIOER, China) Detection System, with relative quantification determined via the 2^−ΔΔCT method.

Western blot

Protein expression was assessed via Western blot analysis. Cell lysates were prepared using radioimmunoprecipitation assay (RIPA) buffer, followed by centrifugation for supernatant collection. Denatured protein extracts (15μg) were resolved by 10% SDS-PAGE electrophoresis and subsequently transferred to polyvinylidenedifluoride (PVDF) membranes. Following blocked with BSA solution (2%), membranes were sequentially probed with specific primary antibodies and corresponding secondary antibodies (Supplementary Table 4). Immunoreactive bands were visualized using Image Studio and quantified via Quantity One Software.

CO-IP

Primary mBMMs from distinct experimental groups underwent osteoclastic induction with M-CSF/RANKL for 5 days, followed by lysis in buffer containing 1 mM PMSF, 1 mM dithiothreitol (DTT), and Protease Inhibitor Cocktail. Cell lysates were subjected to immunoprecipitation with IgG, anti-DCSTAMP, anti-RAP1A, or anti-RAP1B antibodies overnight at 4°C, followed by protein A/G-bead conjugation for 3 hours at 4°C. The immunoprecipitated complexes underwent five washing cycles with protease inhibitor-supplemented PBS at 4°C before either mass spectrometric analysis or resolution by 10% SDS-PAGE followed by immunoblot analysis.

Mass spectrum

Immunoprecipitated protein bands underwent sequential processing for proteomic analysis.

Following extensive washing steps, proteins were subjected to tryptic digestion in acetonitrile solution, followed by purification protocols. Mass spectrometric analysis was performed using a ThermoFisher Q Exactive instrument (ThermoFisher, USA) equipped with a Nano Flex ion source. Protein identification and quantification from MS/MS data were conducted using PEAKS Studio 8.5 analytical platform.

Reference

1 Idris, A. *Bone Research Protocols: Third Edition. Aymen I. Idris (Editor)*. (2019).

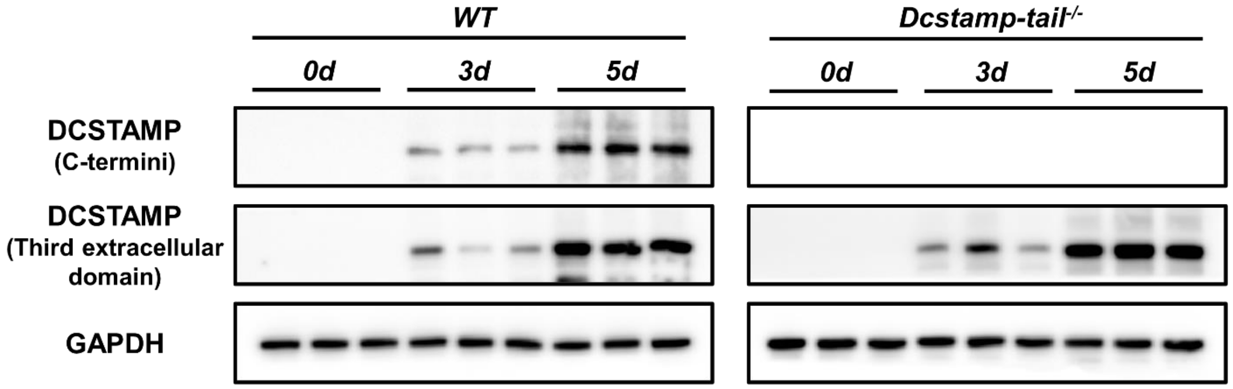

Supplementary Fig. 1. Western blot analysis demonstrating differential expression profiles of discrete DCSTAMP protein domains in primary BMMs isolated from WT and Dcstamp-tail-/- murine models during sequential stages of osteoclastogenic differentiation. Two distinct primary antibodies directed against DCSTAMP were employed, specifically recognizing epitopes within the C-terminal region and the third extracellular domain of this transmembrane glycoprotein, respectively.

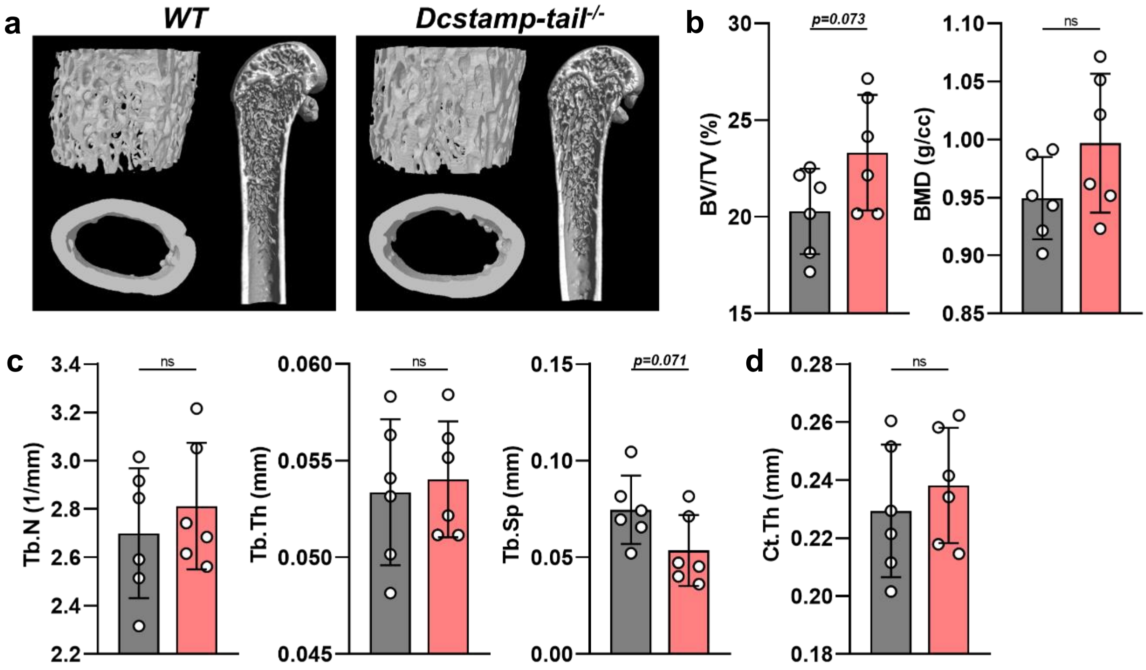

a
WT
Dcstamp-tail$^{-/-}$
b
BV/TV (%)
p=0.073
BMD (g/cc)
ns
c
Tb.N (1/mm)
ns
Tb.Th (mm)
ns
Tb.Sp (mm)
p=0.071
d
Ct.Th (mm)
ns

Supplementary Fig. 2. The bone phenotype of male 12-week-old WT and Dcstamp-tail$^{-/-}$ mice. a. Representative μCT analysis of the distal femur. **b-d.** Calculations from μCT of BV/TV, BMD (b), Tb.N, Tb.Th, Tb.Sp (c) and Ct.Th (d). N = 6 per experimental group. Data are presented as mean ± SD with individual data points depicted as dots. Statistical significance levels are denoted as follows: *$p < 0.05$, ** $p < 0.01$ and *** $p < 0.001$; ns: not significant.

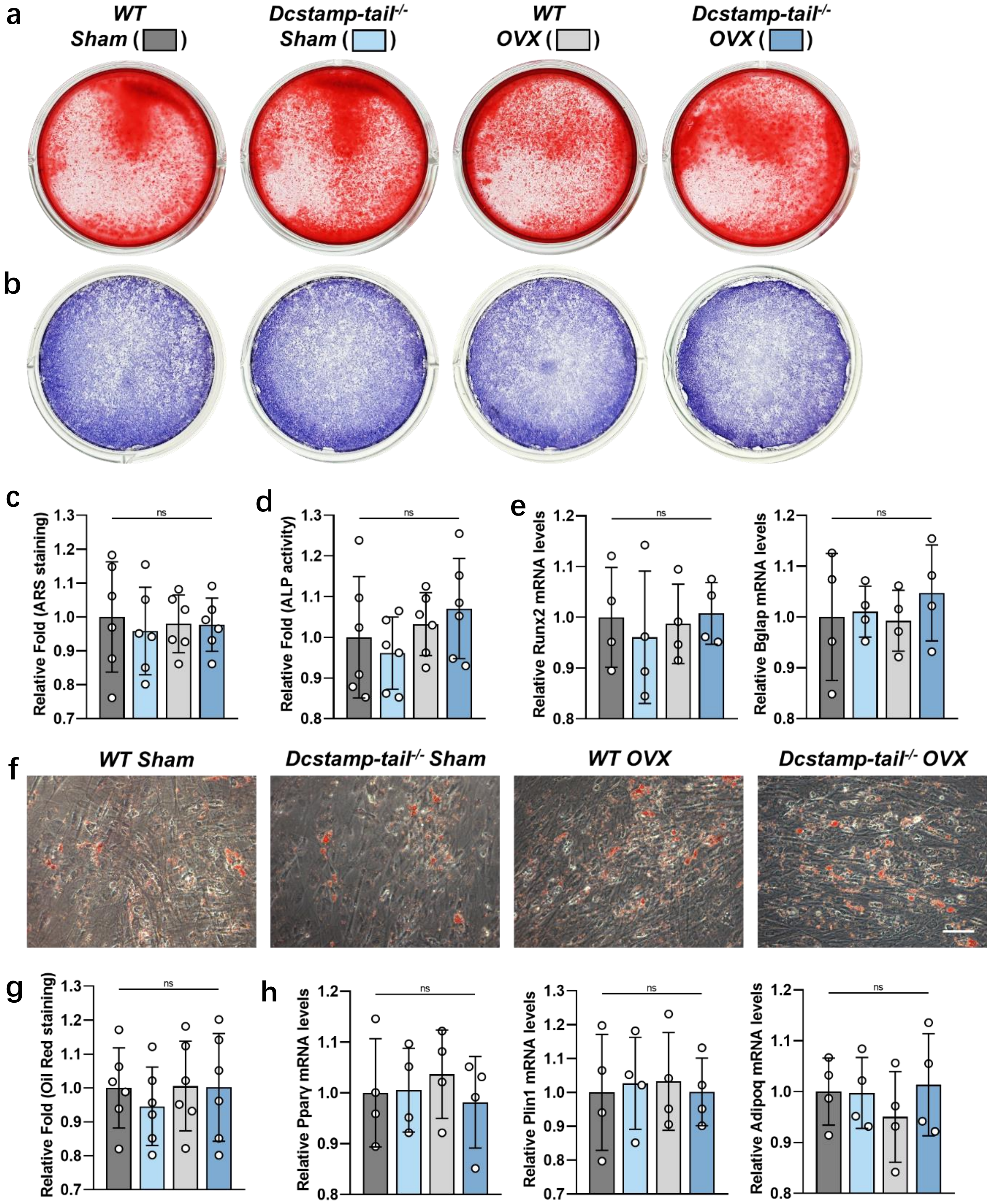

Supplementary Fig. 3. The in vitro osteogenesis and adipogenesis of female WT and Dcstamp-tail$^{-/-}$ mice received Sham or OVX surgery. a. ARS staining of osteoblastic induced primary BMSCs. **b.** ALP staining of osteoblastic induced primary BMSCs. **c.** Quantification of ARS staining. **d.** Quantification of ALP staining. **e.** Transcriptional expression levels of osteogenic genes including Runx2 and Bglap. **f.** Oil O red staining of adipogenic induced

primary BMSCs. (Scale bar: 100μm) **g.** Quantification of Oil O Red staining. **h.** Transcriptional expression levels of adipogenic genes including Ppar, Plin1 and Adipoq. N = 4 in qPCR assays per group; N = 6 in other panels. Data are presented as mean ± SD with individual data points depicted as dots. Statistical significance levels are denoted as follows: *$p < 0.05$, ** $p < 0.01$ and *** $p < 0.001$; ns: not significant.

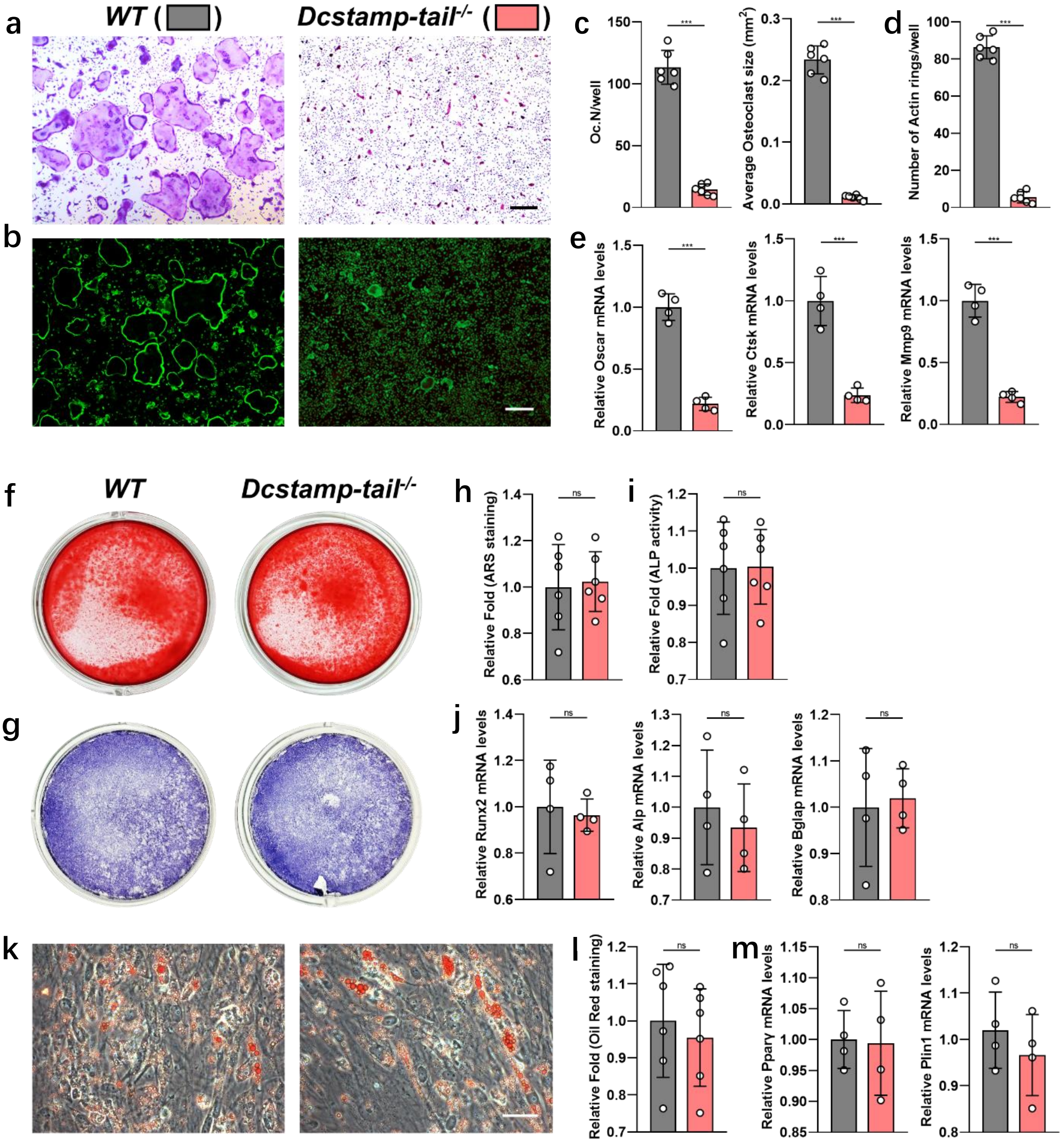

Supplementary Fig. 4. The in vitro osteoclastogenesis, osteogenesis and adipogenesis of male WT and Dcstamp-tail$^{-/-}$ mice. a. TRAP staining mature osteoclasts differentiated from primary BMMs. (Scale bar: 200μm) **b.** Visualization of F-actin cytoskeleton via FITC-phalloidin staining. (Scale bar: 200μm) **c.** Quantification of osteoclast numbers and average size per well from TRAP staining of in vitro differentiated osteoclasts. **d.** Quantification of F-actin rings from FITC-phalloidin staining. **e.** Transcriptional expression levels of osteoclastic genes including Oscar, Ctsk and Mmp9. **f.** ARS staining of osteoblastic induced primary

BMSCs. **g.** ALP staining of osteoblastic induced primary BMSCs. **h.** Quantification of ARS staining. **i.** Quantification of ALP staining. **j.** Transcriptional expression levels of osteogenic genes including Runx2, Alp and Bglap. **k.** Oil O red staining of adipogenic induced primary BMSCs. (Scale bar: 100μm) **l.** Quantification of Oil O Red staining. m. Transcriptional expression levels of adipogenic genes including Pparγ and Plin1. N = 4 in qPCR assays per group; N = 6 in other panels. Data are presented as mean ± SD with individual data points depicted as dots. Statistical significance levels are denoted as follows: *$p < 0.05$, ** $p < 0.01$ and *** $p < 0.001$; ns: not significant.

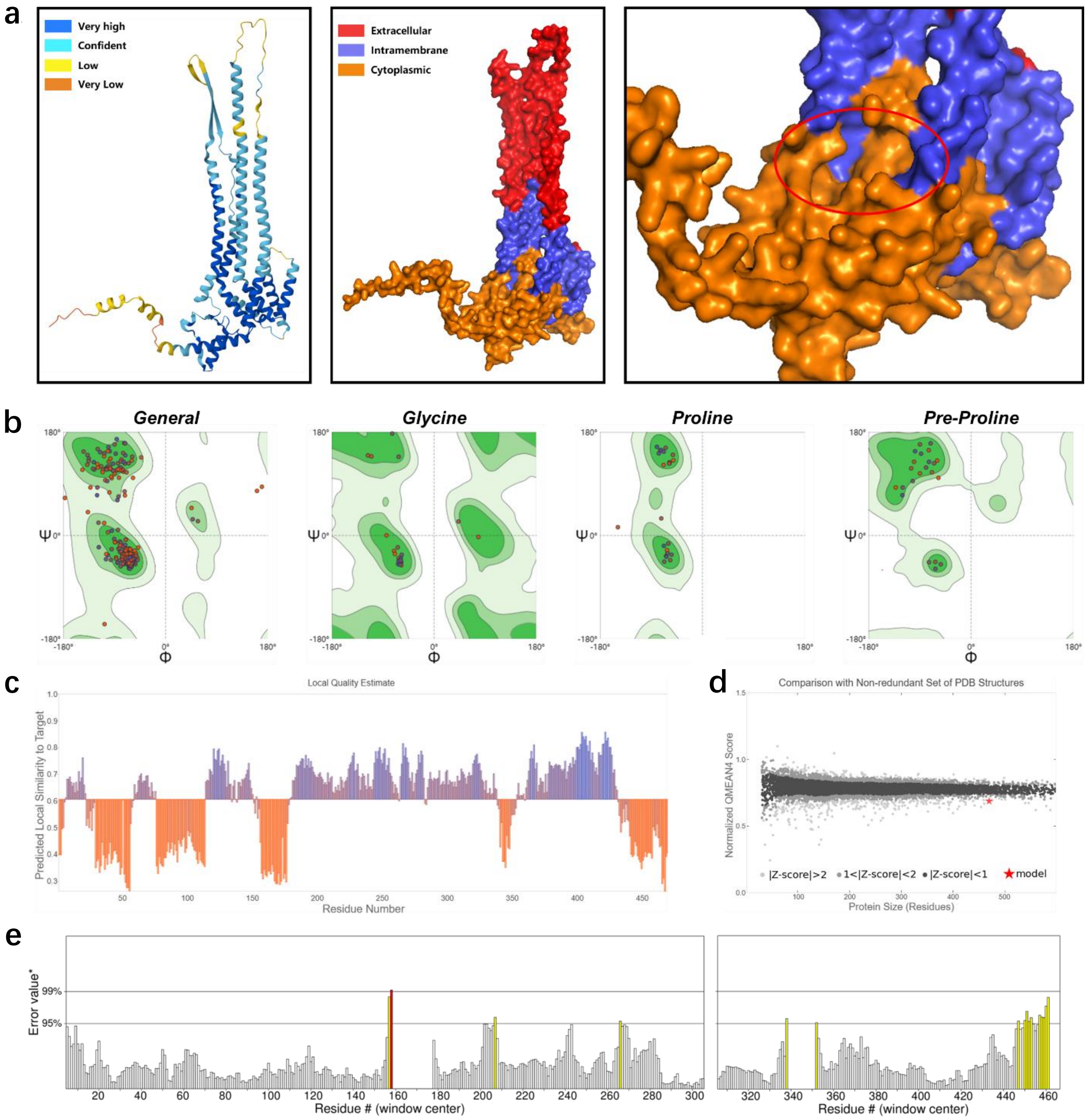

Supplementary Fig. 5. a. The predicted mouse DCSTAMP protein structure by Alphafold and the subcellular distribution of human DCSTAMP protein. Red frame shows possible active site. **b.** Ramachandran plot of human DCSTAMP protein structure using swiss-model structure assessment. **c.** QMEAN local scores of DCSTAMP protein residues in the B-factor column. **d.** Comparison with Non-redundant Set of PDB Structures. **e.** ERRAT analysis showing the percentage of the protein for which the calculated error value falls below the 95% rejection limit.

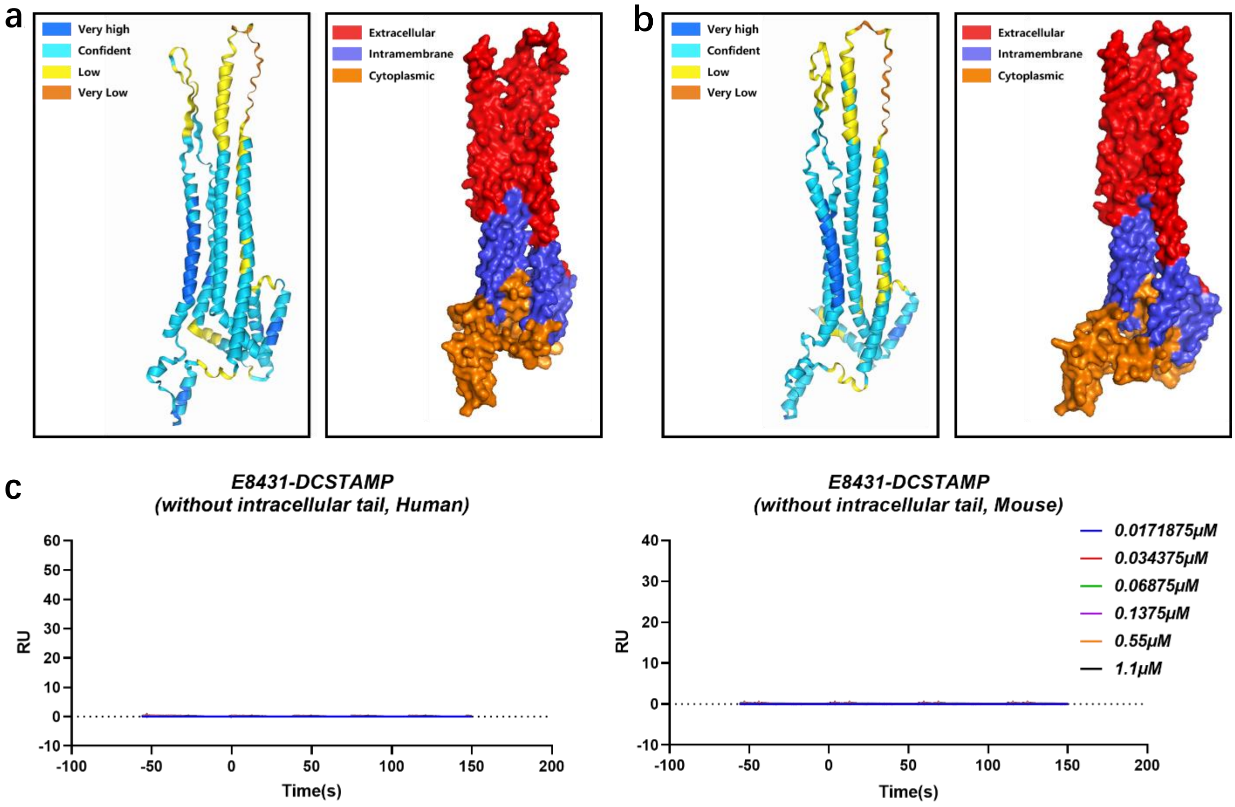

Supplementary Fig. 6. a,b. The predicted structure and subcellular distribution of human DCSTAMP protein without endoplasmic tail (a) and mouse DCSTAMP protein without endoplasmic tail (b). **c.** Sensorgrams from Surface Plasmon Resonance (SPR) experiments depicting the interaction between varying concentrations of E8431 and human/mouse DCSTAMP proteins without intracellular domain.

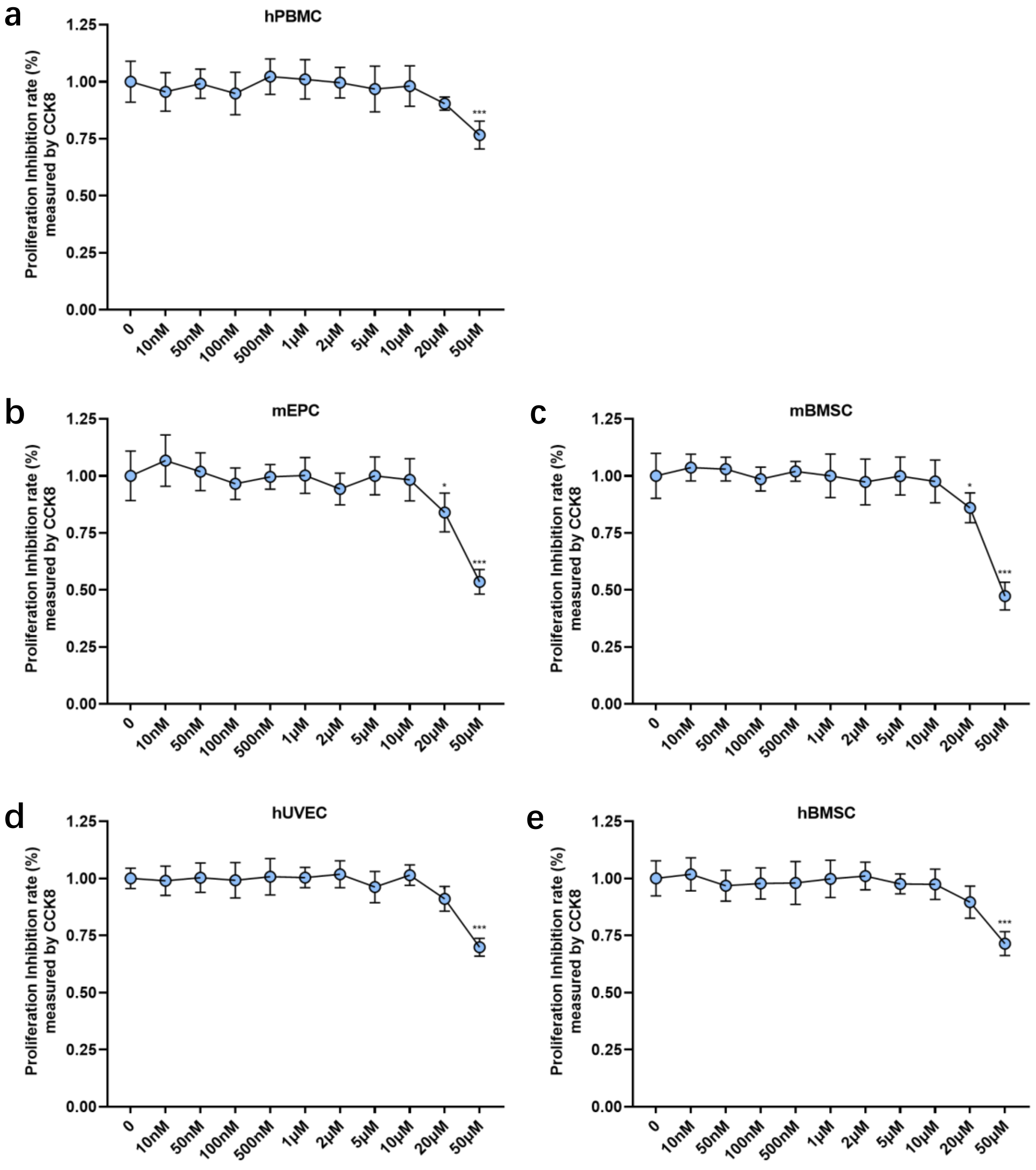

Supplementary Fig. 7. a-e. The proliferation rate under the treatment of various concentrations of E8431 assessed by CCK8 assay. hPBMC (**a**), mEPC (**b**), mBMSC (**c**), Huvec (**d**), and hBMSC (**e**). N = 5 per experimental group. Data are presented as mean ± SD. Statistical significance levels are denoted as follows: *$p < 0.05$, ** $p < 0.01$ and *** $p < 0.001$; ns: not significant.

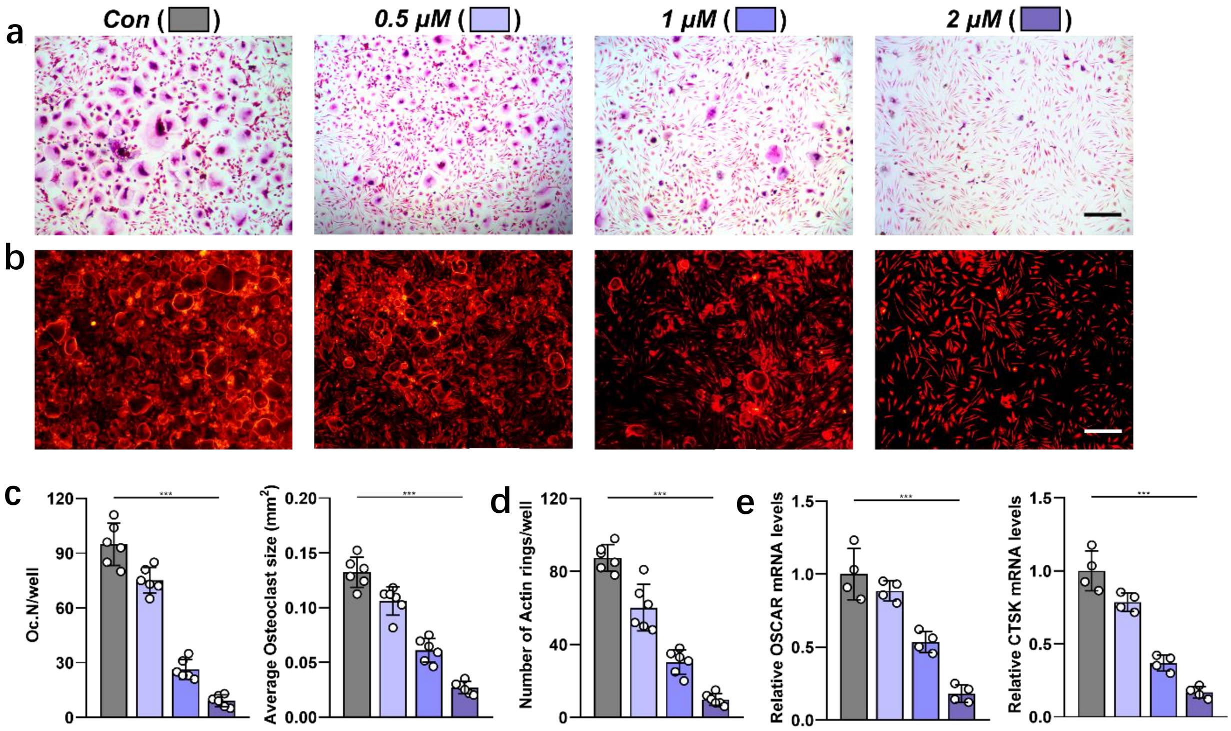

Supplementary Fig. 8. The effects of different concentration of E8431 on osteoclastogenesis of primary hPBMC. a. TRAP staining mature osteoclasts differentiated from primary BMMs. (Scale bar: 200μm) **b.** Visualization of F-actin cytoskeleton via FITC-phalloidin staining. (Scale bar: 200μm) **c.** Quantification of osteoclast numbers and average size per well from TRAP staining of in vitro differentiated osteoclasts. **d.** Quantification of F-actin rings from FITC-phalloidin staining. **e.** Transcriptional expression levels of osteoclastic genes including OSCAR and CTSK. N = 4 in qPCR assays per group; N = 6 in other panels. Data are presented as mean ± SD with individual data points depicted as dots. Statistical significance levels are denoted as follows: *$p < 0.05$, ** $p < 0.01$ and *** $p < 0.001$; ns: not significant.

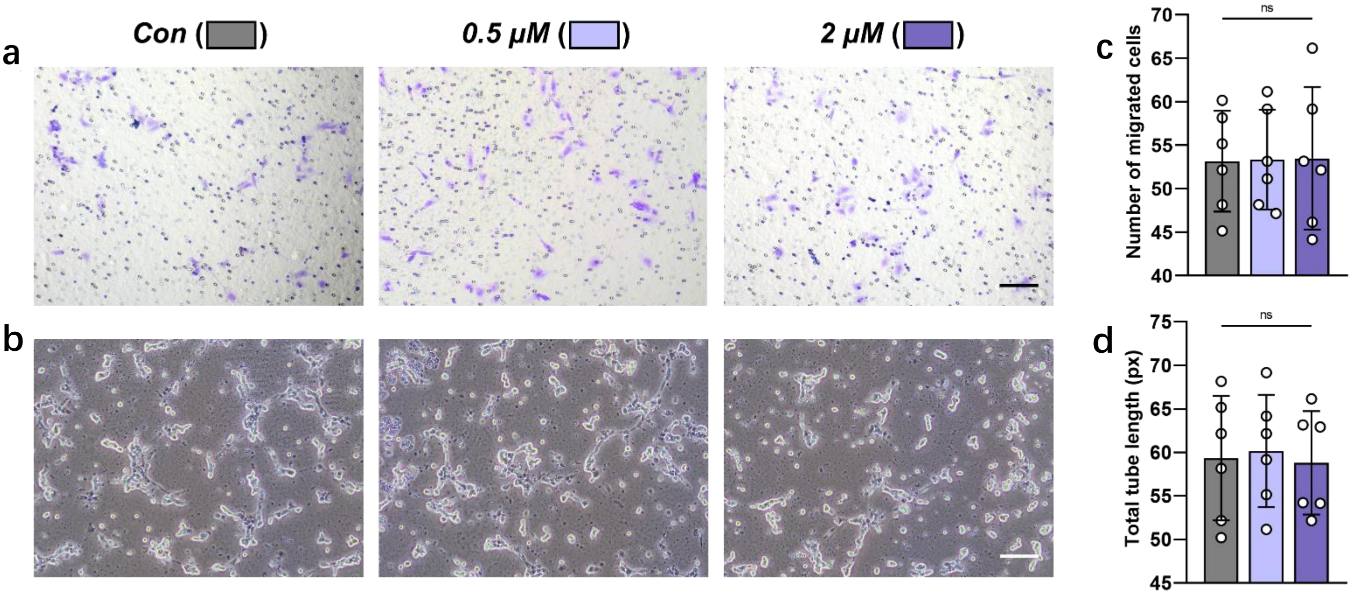

Supplementary Fig. 9. E8431 exerts no direct effects on angiogenesis of mouse EPCs in vitro. a, c. EPC migration capacity measured by the transwell migration assay. (Scale bar: 200μm) **b, d.** Tube formation and quantification of tube length of EPCs on Matrigel. (Scale bar: 200μm) N = 6 per experimental group. Data are presented as mean ± SD with individual data points depicted as dots. Statistical significance levels are denoted as follows: *$p < 0.05$, ** $p < 0.01$ and *** $p < 0.001$; ns: not significant.

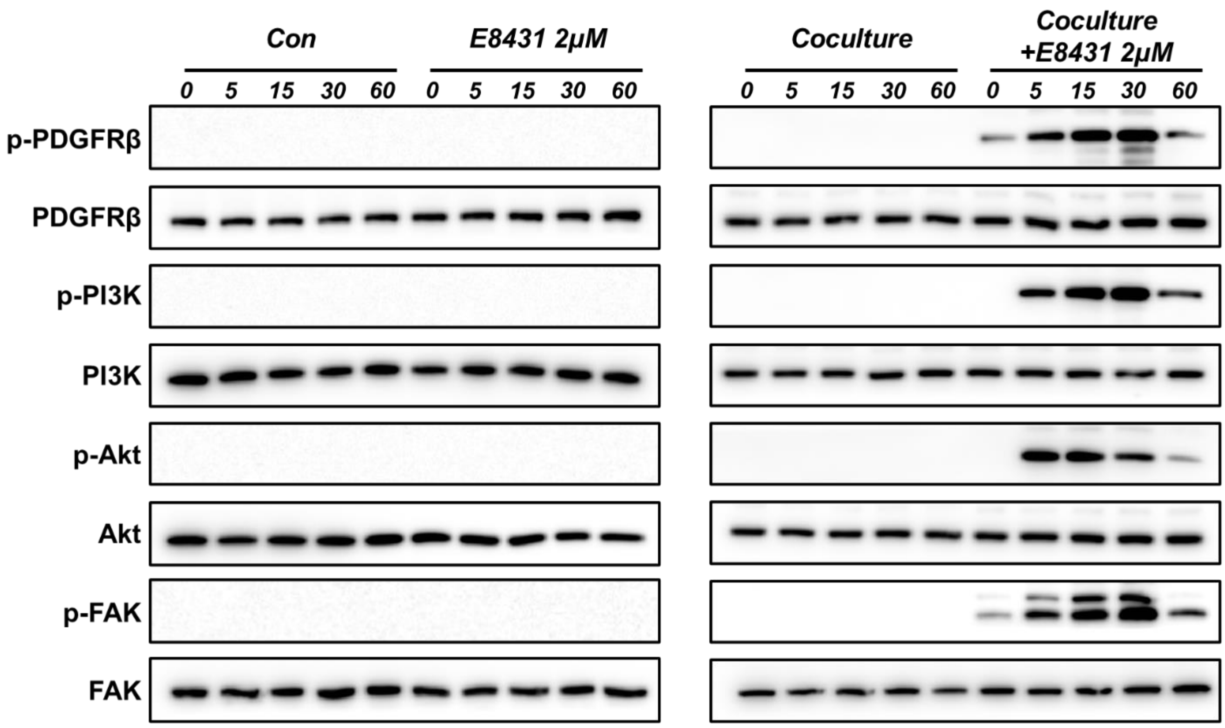

Supplementary Fig. 10. Western blot analysis of the phosphorylation of PDGFRβ, PI3K, Akt and FAK in mouse EPCs subjected to varying experimental conditions: negative controls, exposure to 2μM E8431, coculture system implementation, and combined coculture with 2μM E8431 supplementation.

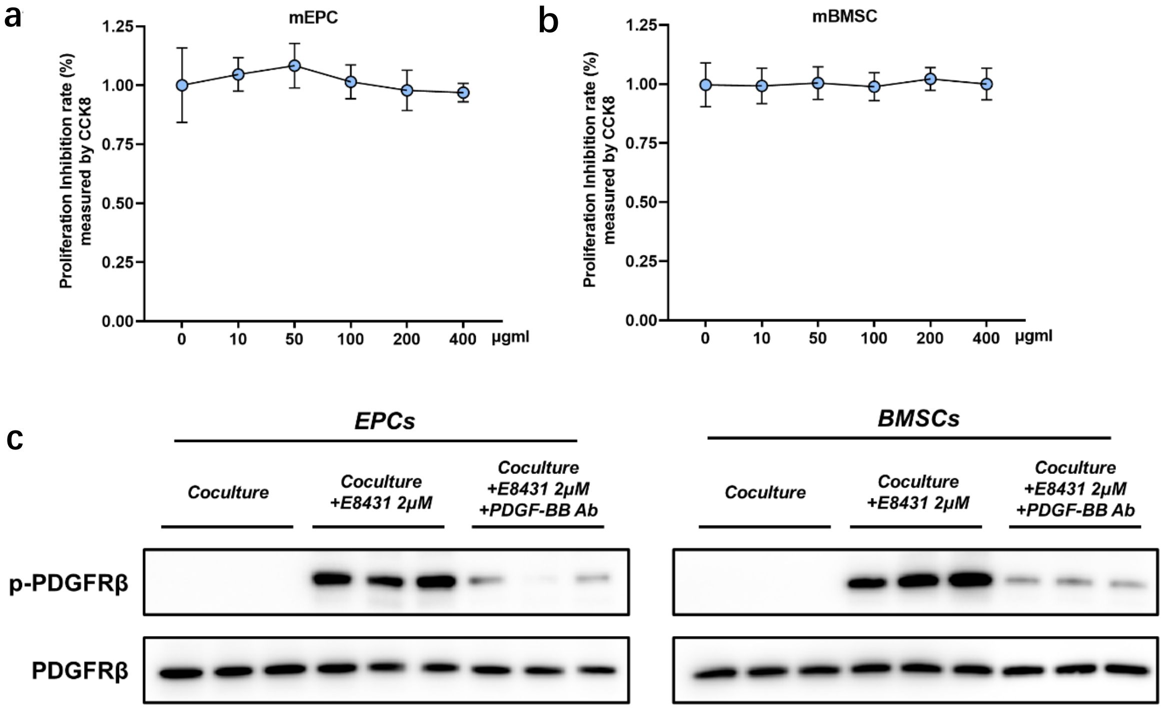

Supplementary Fig. 11. a, b. The proliferation rate under the treatment of various concentrations of PDGF-BB neutralization antibody assessed by CCK8 assay in mEPC and mBMSC. N = 5 per experimental group. **c.** Western blot analysis of the phosphorylation of PDGFRβin mouse EPCs and BMSCs co-cultured with BMMs under differential treatment regimens: negative control, 2μM E8431 monotherapy, and combined administration of 2μM E8431 with PDGF-BB neutralizing antibody (100μg/ml) for a 30-minute exposure period. Data are presented as mean ± SD. Statistical significance levels are denoted as follows: *$p < 0.05$, ** $p < 0.01$ and *** $p < 0.001$; ns: not significant.

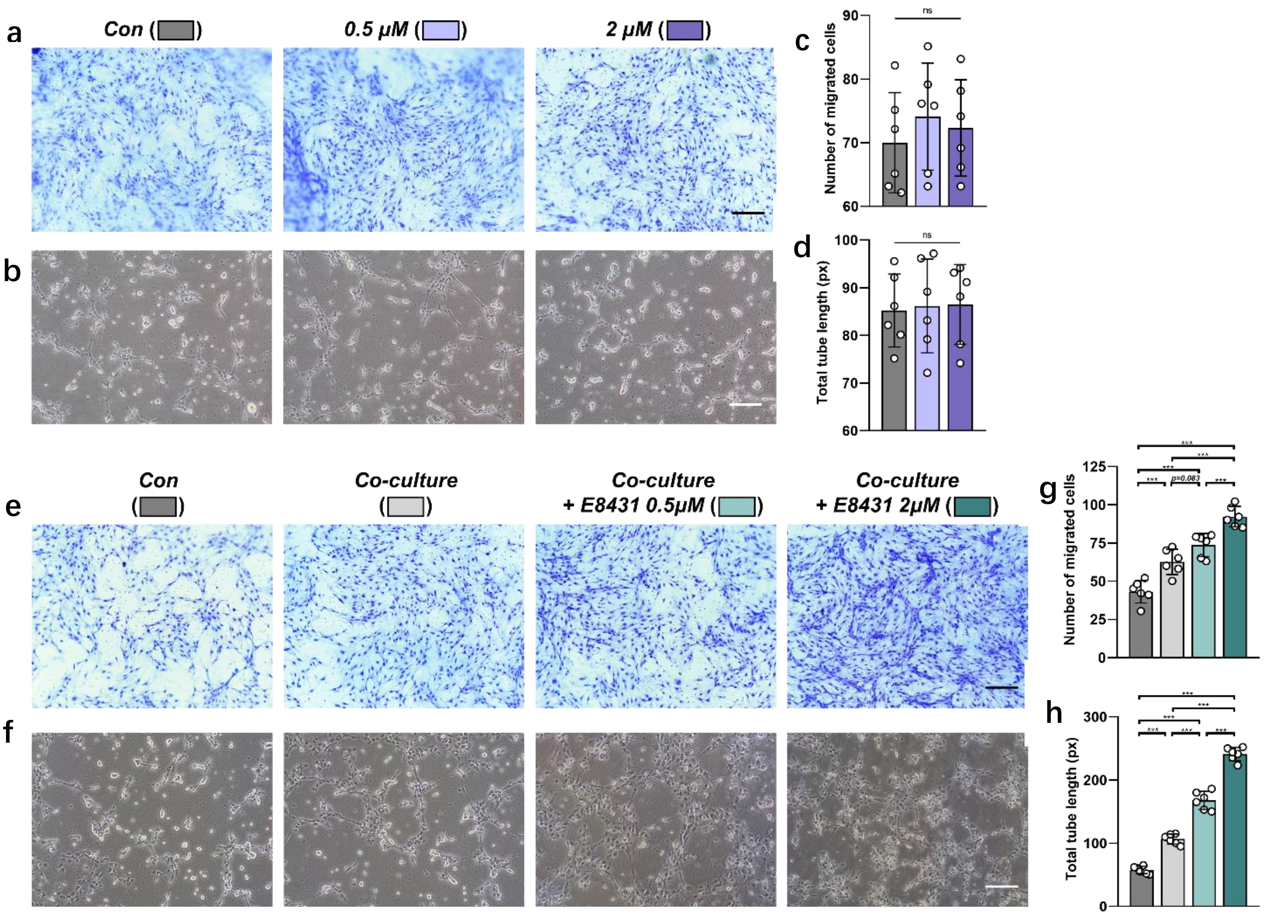

Supplementary Fig. 12. E8431 promotes angiogenesis of hUVEC via suppression of osteoclastogenesis of osteoclasts. a, c. EPC migration capacity measured by the transwell migration assay of E8431 directly treated hUVEC. (Scale bar: 200μm) **b, d.** Tube formation and quantification of tube length of EPCs on Matrigel of E8431 directly treated hUVEC. (Scale bar: 200μm) **e, g.** EPC migration capacity measured by the transwell migration assay in co-cultured hUVEC. (Scale bar: 200μm) **f, h.** Tube formation and quantification of tube length of EPCs on Matrigel in co-cultured hUVEC. (Scale bar: 200μm) N = 6 per experimental group. Data are presented as mean ± SD with individual data points depicted as dots. Statistical significance levels are denoted as follows: *$p < 0.05$, ** $p < 0.01$ and *** $p < 0.001$; ns: not significant.

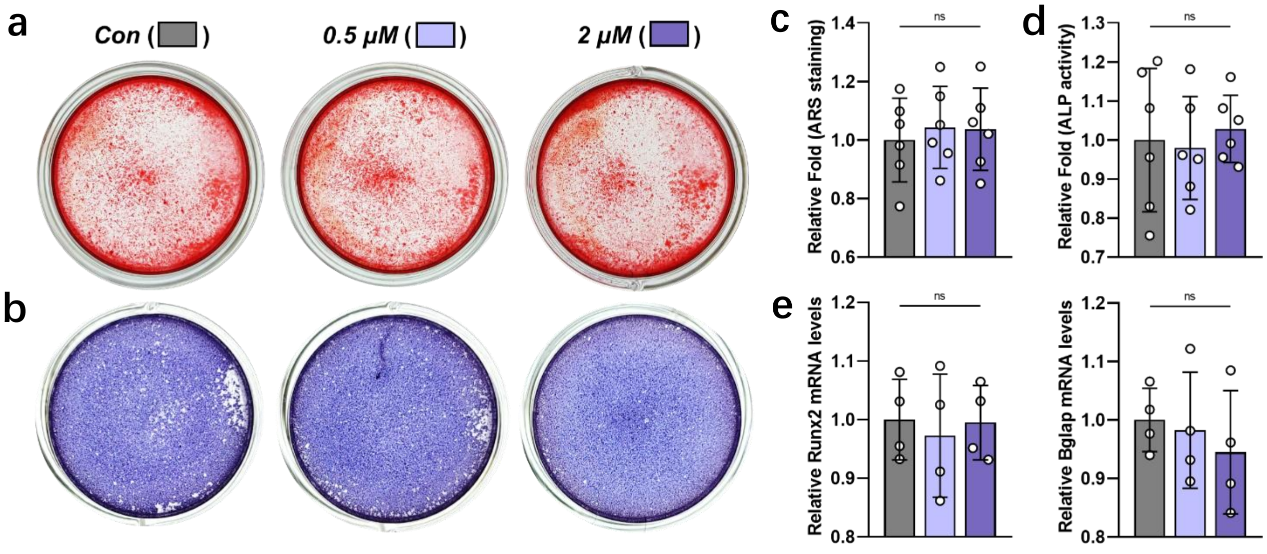

Supplementary Fig. 13. The effects of different concentration of E8431 on osteogenesis of primary mouse BMSC. a. ARS staining of osteoblastic induced primary BMSCs. **b.** ALP staining of osteoblastic induced primary BMSCs. **c.** Quantification of ARS staining. **d.** Quantification of ALP staining. **e.** Transcriptional expression levels of osteogenic genes including Runx2 and Bglap. N = 4 in qPCR assays per group; N = 6 in other panels. Data are presented as mean ± SD with individual data points depicted as dots. Statistical significance levels are denoted as follows: *$p < 0.05$, ** $p < 0.01$ and *** $p < 0.001$; ns: not significant.

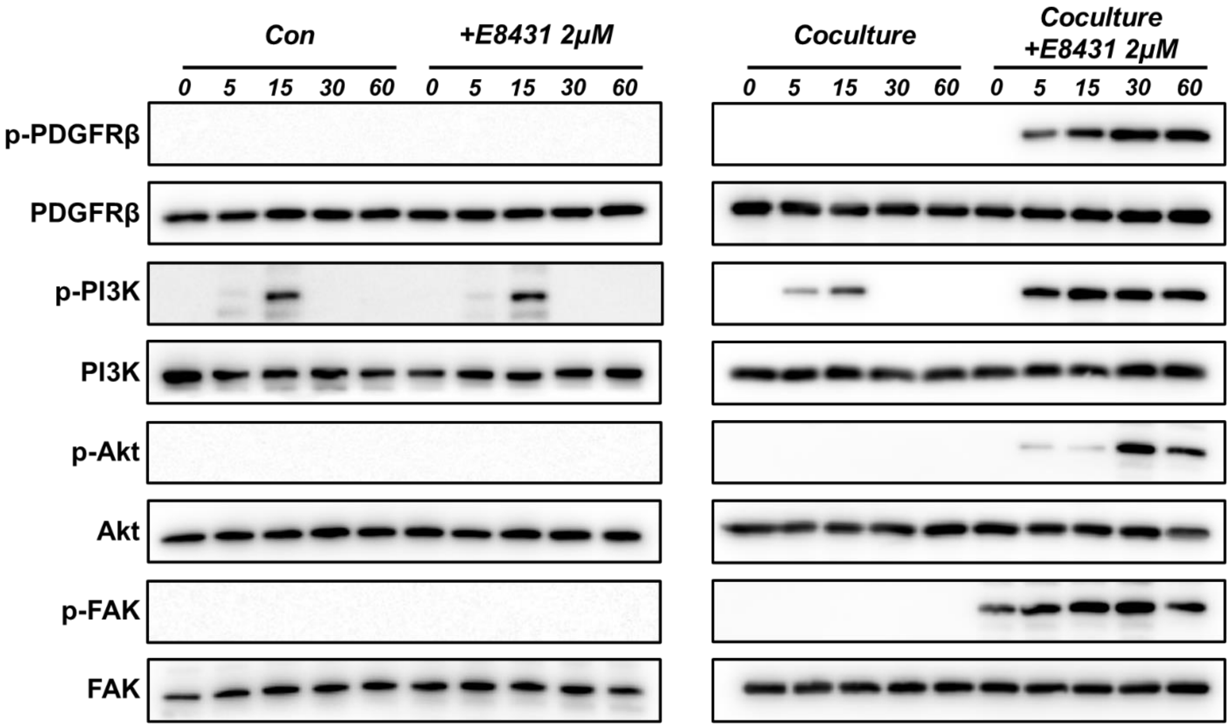

Supplementary Fig. 14. Western blot analysis of the phosphorylation of PDGFRβ, PI3K, Akt and FAK in mouse BMSCs subjected to varying experimental conditions: negative controls, exposure to 2μM E8431, coculture system implementation, and combined coculture with 2μM E8431 supplementation.

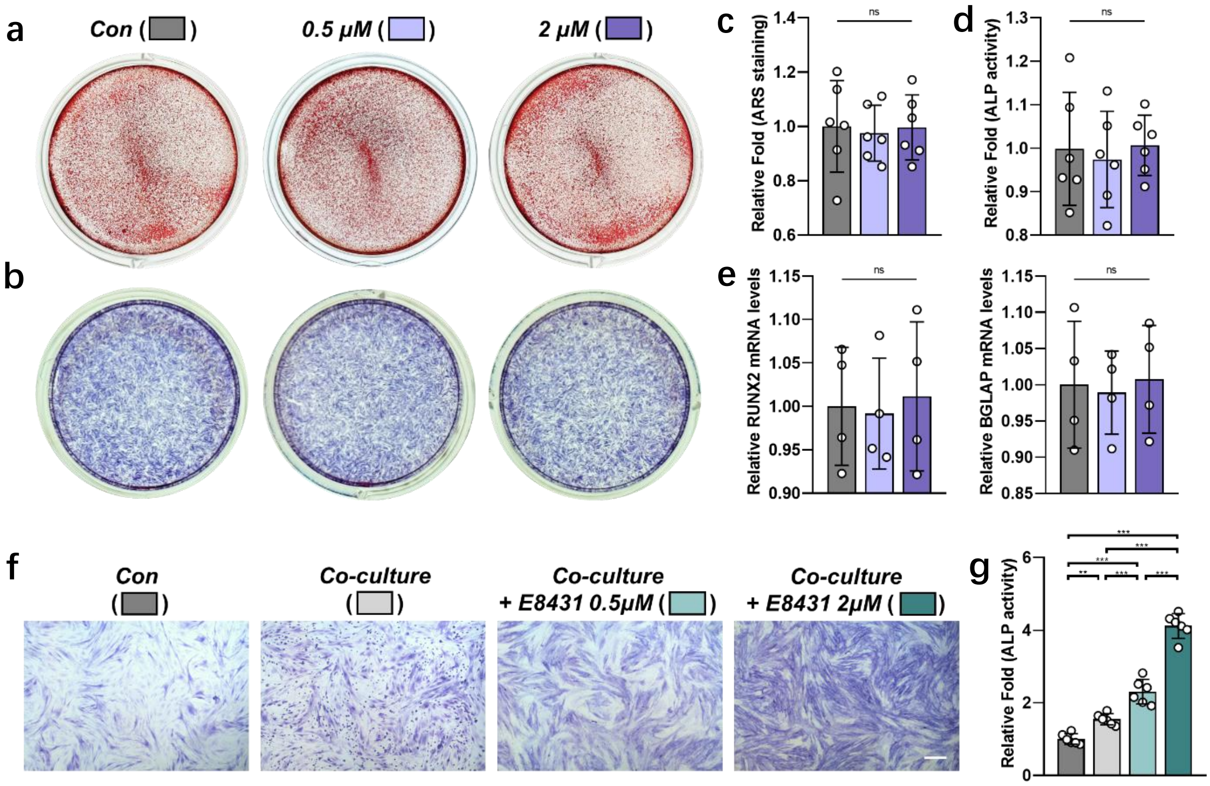

Supplementary Fig. 15. E8431 promotes osteogenesis of hBMSC via suppression of osteoclastogenesis of osteoclasts. a. ARS staining of osteoblastic induced hBMSCs. **b.** ALP staining of osteoblastic induced hBMSCs. **c.** Quantification of ARS staining. **d.** Quantification of ALP staining. **e.** Transcriptional expression levels of osteogenic genes including RUNX2 and BGLAP. **f, g.** ALP staining and quantification of osteoblastic induced co-cultured hBMSCs. (Scale bar: 200μm) N = 4 in qPCR assays per group; N = 6 in other panels. Data are presented as mean ± SD with individual data points depicted as dots. Statistical significance levels are denoted as follows: *$p < 0.05$, ** $p < 0.01$ and *** $p < 0.001$; ns: not significant.

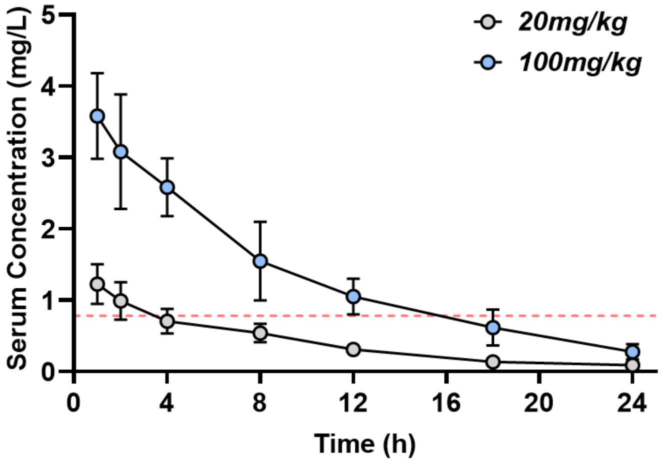

Supplementary Fig. 16. The serum concentrations of E8431 from 1 to 24 hours after the intraperitoneal injection of E8431 measured by mass spectrum. The red dotted line showed suitable concentration of E8431: 2μM. N = 6 per experimental group. Data are presented as mean ± SD. Statistical significance levels are denoted as follows: **p* < 0.05, ** *p* < 0.01 and *** *p* < 0.001; ns: not significant.

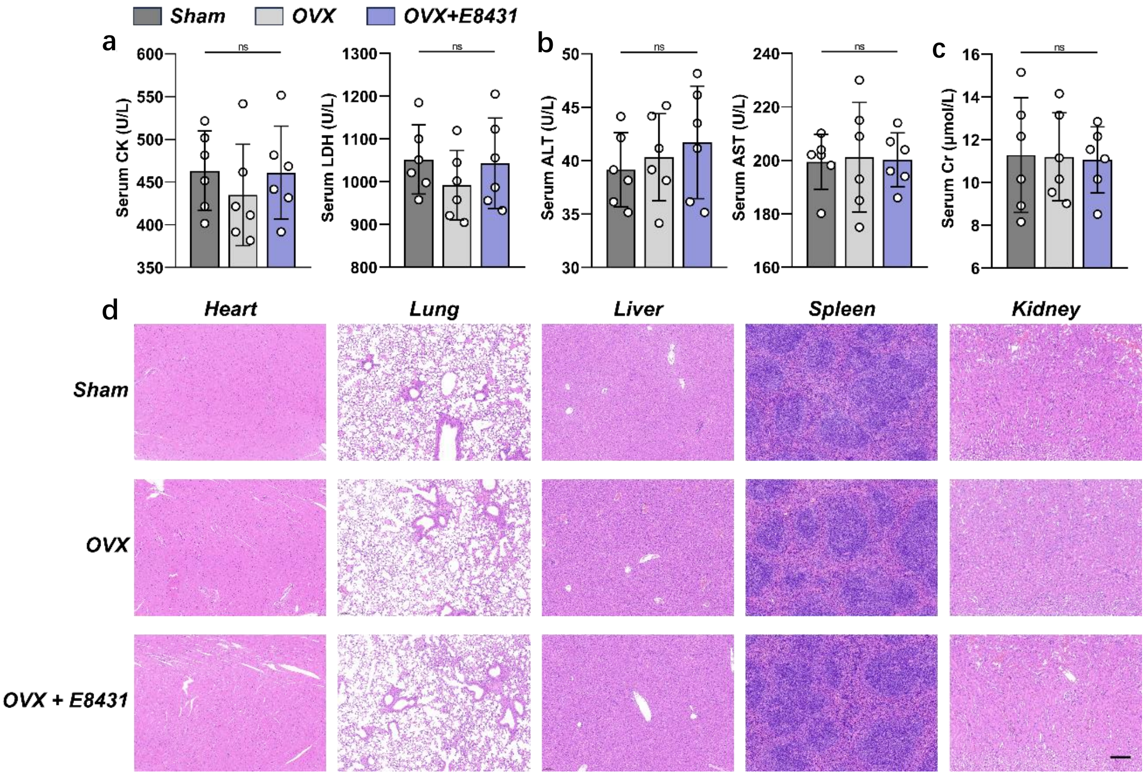

Supplementary Fig. 17. The toxicity of six-week treated E8431 in mice. a. Serum creatine kinase (CK) and lactate dehydrogenase (LDH) levels. **b.** Serum alanine transaminase (ALT) and aspartate aminotransferase (AST) levels. **c.** Serum creatinine levels. **d.** HE staining of sections of heart, lung, liver, spleen and kidney. (Scale bar: 200μm) N = 6 per experimental group. Data are presented as mean ± SD with individual data points depicted as dots. Statistical significance levels are denoted as follows: *$p < 0.05$, ** $p < 0.01$ and *** $p < 0.001$; ns: not significant.

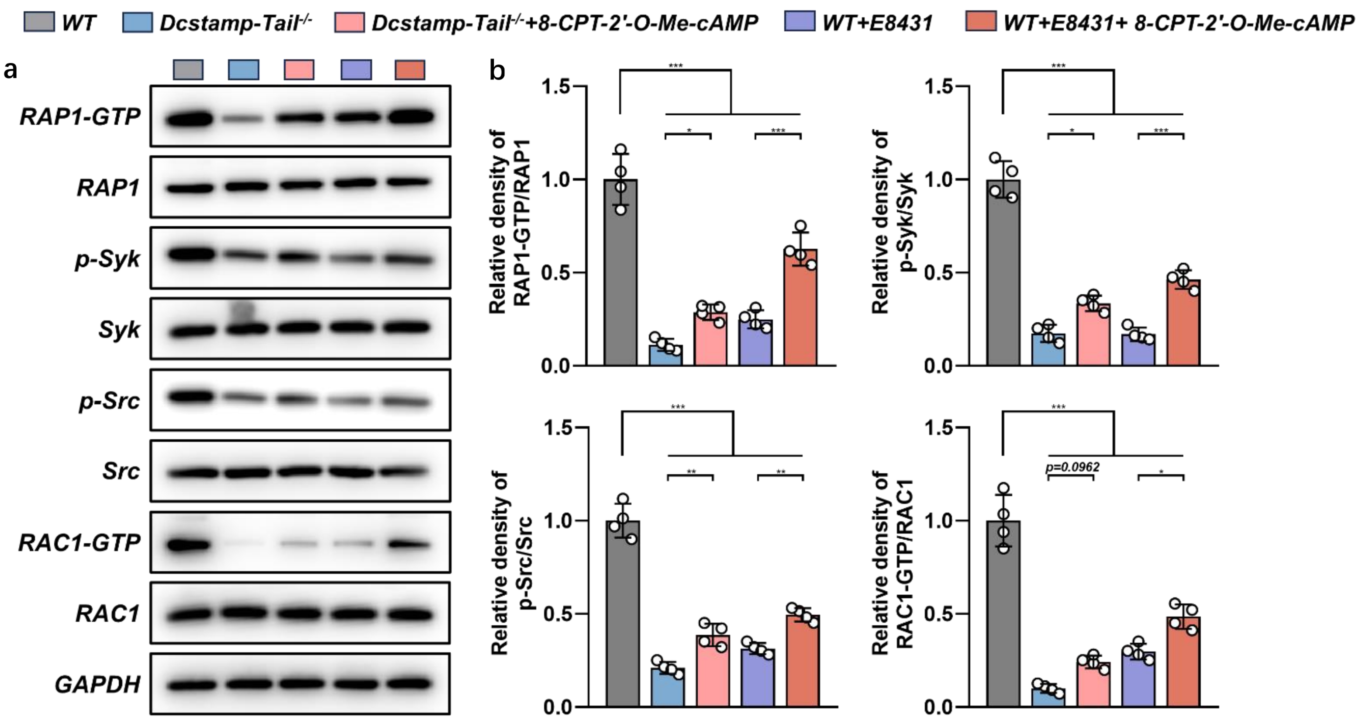

Supplementary Fig. 18. a. Western blot and corresponding quantification measuring the protein levels of RAP1-GTP, RAP1, phosphorylated Syk (p-Syk), Syk, phosphorylated Src (p-Src), Src, RAC1-GTP, RAC1 and GAPDH in 8-CPT-2'-O-Me-cAMP treated or control groups. b. Quantification of the Western blot results, with N=4 samples analyzed. Data are presented as mean ± SD with individual data points depicted as dots. Statistical significance levels are denoted as follows: *$p < 0.05$, ** $p < 0.01$ and *** $p < 0.001$; ns: not significant.

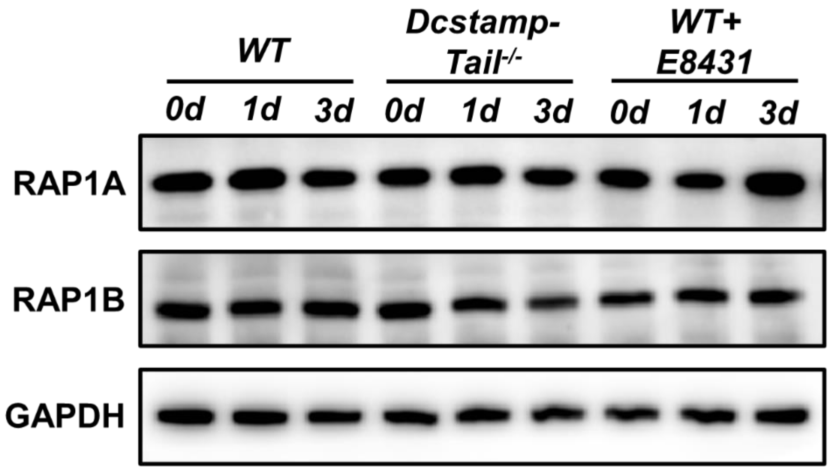

Supplementary Fig. 19. Western blot analysis measuring the protein levels of RAP1A and RAP1B during osteoclastogenesis from primary mBMMs obtained from wild-type (WT), Dcstamp tail$^{-/-}$, and WT mice treated with E8431.

Supplementary Table 1

Sequences of the primers used in mouse genotyping

Forward 5’- CCTGGTTACACACAGCTACTT -3’

Reverse 5’- AATGGCTGCTTTGATCGTTTC -3’

Supplementary Table 2

Sequences of the products in mouse genotyping of WT and Dcstamp-tail$^{-/-}$ mice

WT:

CCTGGTTACACACAGCTACTTTAGACAAAGCTCCCTGAATATTTTCAGGAAAATAG
CATGTTTCAAAGAAGTAGAAAGACAGTCAGATGAGACAGAGTAGAAGATGGCGTA
CATAGTGATTATTTTATTCTTTCATTTTAGAAGCAAGGAACCCAAGGAGTCGTCCAT
GATTCTGCCTTTAATATATCTATGTTTGAACCGAGCTGCATTCCTAAACCACGTCTC
AGTGTGTCTGAGACTTGGGTTCCTCTCAGTATTATTCTGTTAACACTAATAATACTA
GGATTGTTGTCTTCTATGCTGATGCAGCTTAAAATTCTCGTGTCAGTCTCCTTCTAC
CCCAAAGTGGAGAGGGAGAGAATTGAATACCTGCATGCGAAGCTCCTTGAGAAAC
GATCAAAGCAGCCATT

Dcstamp-tail$^{-/-}$:

CCTGGTTACACACAGCTACTTTAGACAAAGCTCCCTGAATATTTTCAGGAAAATAG
CATGTTTCAAAGAAGTAGAAAGACAGTCAGATGAGACAGAGTAGAAGATGGCGTA
CATAGTGATTATTTTATTCTTTCATTTTAGAAGCAAGGAACCCAAGGAGTCGTCCAT
GATTCTGCCTTTAATATATCTATGTTTGAACCGAGCTGCATTCCTAAACCACGTCTC
AGTGTGTCTGAGACTTGGGTTCCTCTCAGTATTATTCTGTTAACACTAATAATACTA
GGATTGTTGTCTTCTATGCTTGCAGCTTAAAATTCTCGTGTCAGTCTCCTTCTACCC
CAAAGTGGAGAGGGAGAGAATTGAATACCTGCATGCGAAGCTCCTTGAGAAACG
ATCAAAGCAGCCATT

Supplementary Table 3

Sequences of the primers used in RT-qPCR

Gene Name	Primers
Ctsk	Forward 5'- AGTAGCCACGCTTCCTATCC -3'<br>Reverse 5'- CCATGGGTAGCAGCAGAAAC -3'
Acp5	Forward 5'- ATGGGCGCTGACTTCATCAT -3'<br>Reverse 5'- GGTCTCCTGGAACCTCTTGT -3'
Oscar	Forward 5'- CCTAGCCTCATACCCCCAG -3'<br>Reverse 5'- CGTTGATCCCAGGAGTCACAA -3'
Mmp9	Forward 5'- CTGGACAGCCAGACACTAAAG -3'<br>Reverse 5'- CTCGCGGCAAGTCTTCAGAG -3'
Runx2	Forward 5'- GCTCTTCCCAAAGCCAGAGT -3'<br>Reverse 5'- GGATCCTGACGAAGTGCCAT -3'
Alp	Forward 5'- GAATCTTCCCCAAGGGCCAA -3'<br>Reverse 5'- CAGAATGTTCCACGGAGGCT -3'
Bglap	Forward 5'- GCAATAAGGTAGTGAACAGACTCC -3'<br>Reverse 5'- CCATAGATGCGTTTGTAGGCGG -3'
Pparγ	Forward 5'- TCGTGGTACTTTACGCCTCG -3'<br>Reverse 5'- ACTCCCCGTTTCTGACTCCT -3'
Plin1	Forward 5'- CTGTGTGCAATGCCTATGAGA -3'<br>Reverse 5'- CTGGAGGGTATTGAAGAGCCG -3'
Adipoq	Forward 5'- ATGACGACTGCCATCCTAGAG -3'<br>Reverse 5'- GCTCCCTAAAGAGCTGGGG -3'
OSCAR	Forward 5'- ACTCTGGCTCCTCCGACTACAC -3'<br>Reverse 5'- TCCGCCACTCAGGTTGGAAGT -3'
CTSK	Forward 5'- ACTCAAAGTACCCCTGTCTCAT -3'<br>Reverse 5'- CCACAGAGCTAAAAGCCCAAC -3'
RUNX2	Forward 5'- TGGTTACTGTCATGGCGGGTA -3'<br>Reverse 5'- TCTCAGATCGTTGAACCTTGCTA -3'
BGLAP	Forward 5'- CACTCCTCGCCCTATTGGC -3'<br>Reverse 5'- CCCTCCTGCTTGGACACAAAG -3'
Gapdh	Forward 5'- AGGTGGTGAAGCAGGCATCTGA -3'<br>Reverse 5'- CGGCATCGAAGGTGGAAGAGTG -3'
GAPDH	Forward 5'- GTCTCCTCTGACTTCAACAGCG -3'<br>Reverse 5'- ACCACCCTGTTGCTGTAGCCAA -3'

Supplementary Table 4. Primary and secondary antibodies used in this study.

Antibodies	Supplier	Catalog Number
Anti-DC-STAMP Antibody, clone 1A2	Sigma-Aldrich	MABF39-I
TM7SF4 Polyclonal Antibody	ThermoFisher	PA5-89951
NeutraKine® PDGF-BB Mouse McAb	Proteintech	69020-1-Ig
Endomucin Antibody (V.7C7)	Santa Cruz Biotechnology	Cat#sc-65495
Rabbit IgG, monoclonal	Abcam	Cat#ab172730
Donkey Anti-Rabbit IgG H&L (Alexa Fluor® 647)	Abcam	Cat#ab150075
PE/Cyanine7 anti-mouse CD31 Antibody	BioLegend	Cat#102417
FITC anti-mouse TER-119/Erythroid Cells Antibody	BioLegend	Cat#116205
FITC anti-mouse CD45 Recombinant Antibody	BioLegend	Cat#157607
CD31 Polyclonal antibody	Proteintech	Cat#28083-1-AP
PDGF-BB Antibody	R&D Systems	Cat#AF-220-NA
Osteocalcin/OCN Polyclonal antibody	Proteintech	Cat#23418-1-AP
Rap1A/Rap1B (26B4) Rabbit mAb	Cell Signaling Technology	Cat#2399
Phospho-Syk (Tyr525/526) (C87C1) Rabbit mAb	Cell Signaling Technology	Cat#2710
Syk Antibody (4D10)	Santa Cruz Biotechnology	Cat#sc-1240
Phospho-Src Family (Tyr416) Antibody	Cell Signaling Technology	Cat#2101
Src (36D10) Rabbit mAb	Cell Signaling Technology	Cat#2109
Anti-Rac1 Antibody, clone 23A8	MilliporeSigma	Cat#05-389
GAPDH	ABclonal	Cat#A22035
Rap 1A Antibody (C-10)	Santa Cruz Biotechnology	Cat#sc-373968
Rap1B (36E1) Rabbit mAb	Cell Signaling Technology	Cat#2326
HRP-conjugated Goat Anti-Rabbit IgG(H+L)	Proteintech	Cat#SA00001-2
HRP-conjugated Goat Anti-Mouse IgG(H+L)	Proteintech	Cat#SA00001-1
PDGF Receptor beta (28E1) Rabbit mAb	Cell Signaling Technology	Cat#3169
Phospho-PDGF Receptor beta (Tyr751) Antibody	Cell Signaling Technology	Cat#3161
Phospho-PI3 Kinase p85 (Tyr458)/p55 (Tyr199) [illegible]	Cell Signaling Technology	Cat#4228
PI3 Kinase p110alpha (C73F8) Rabbit mAb	Cell Signaling Technology	Cat#4249
Phospho-Akt (Ser473) (D9E) XP® Rabbit mAb	Cell Signaling Technology	Cat#4060
Akt Antibody	Cell Signaling Technology	Cat#9272
Phospho-FAK (Tyr397) Antibody	Cell Signaling Technology	Cat#3283
FAK Antibody	Cell Signaling Technology	Cat#3285
